# Co-administration with A1M does not influence apoptotic response of ^177^Lu-octreotate in GOT1 neuroendocrine tumors

**DOI:** 10.1038/s41598-023-32091-9

**Published:** 2023-04-19

**Authors:** Nishte Rassol, Charlotte Andersson, Daniella Pettersson, Amin Al-Awar, Emman Shubbar, Anikó Kovács, Bo Åkerström, Magnus Gram, Khalil Helou, Eva Forssell-Aronsson

**Affiliations:** 1 Department of Medical Radiation Sciences, Institute of Clinical Sciences, Sahlgrenska Academy, University of Gothenburg, Gothenburg, Sweden; 2 Sahlgrenska Center for Cancer Research, Sahlgrenska Academy, University of Gothenburg, Gothenburg, Sweden; 3 Department of Clinical Pathology, Sahlgrenska University Hospital, Gothenburg, Sweden; 4 Department of Clinical Sciences, Infection Medicine, Lund University, Lund, Sweden; 5 Neonatology Unit, Department of Clinical Sciences, Pediatrics, Lund University, Lund, Sweden; 6 Department of Oncology, Institute of Clinical Sciences, Sahlgrenska Academy, University of Gothenburg, Gothenburg, Sweden; 7 Medical Physics and Biomedical Engineering, Sahlgrenska University Hospital, Gothenburg, Sweden

**Keywords:** Cancer, Molecular medicine, Oncology, Physics

## Abstract

Recombinant α_1_-microglobulin (A1M) is a proposed radioprotector during ^177^Lu-octreotate therapy of neuroendocrine tumors (NETs). To ensure a maintained therapeutic effect, we previously demonstrated that A1M does not affect the ^177^Lu-octreotate induced decrease in GOT1 tumor volume. However, the underlying biological events of these findings are still unknown. The aim of this work was to examine the regulation of apoptosis-related genes in GOT1 tumors short-time after i.v. administration of ^177^Lu-octreotate with and without A1M or A1M alone. Human GOT1 tumor-bearing mice received 30 MBq ^177^Lu-octreotate or 5 mg/kg A1M or co-treatment with both. Animals were sacrificed after 1 or 7 days. Gene expression analysis of apoptosis-related genes in GOT1 tissue was performed with RT-PCR. In general, similar expression patterns of pro- and anti-apoptotic genes were found after ^177^Lu-octreotate exposure with or without co-administration of A1M. The highest regulated genes in both irradiated groups compared to untreated controls were FAS and TNFSFRS10B. Administration of A1M alone only resulted in significantly regulated genes after 7 days. Co-administration of A1M did not negatively affect the transcriptional apoptotic response of ^177^Lu-octreotate in GOT1 tumors.

## Introduction

Molecular radionuclide therapy (MRT) is a systemic treatment option for patients with disseminated neuroendocrine (NE) tumors (NETs). Most well-differentiated NETs overexpress membrane-bound somatostatin receptors (SSTRs), which provides systemically available targets for the treatment of NETs with radiolabeled somatostatin analogues^1^. The European Medicines Agency (EMA)^2^ and the Food and Drug Administration (FDA)^3^ approved MRT with ^177^Lu-octreotate (Lutathera) for patients with certain NET types using ^177^Lu in MRT is favorable due to the high emission yield of electrons (78.6%, 148 keV/nuclear transition)^4^ with a relatively short maximum range (1.8 mm), resulting in locally absorbed doses to cells in close proximity, and thus sparing healthy tissues^5^. However, the distribution of the radiopharmaceutical is not restricted only to tumor cells, and bone marrow and kidneys are the most critical organs after ^177^Lu-octreotate therapy. The treatment protocol includes protective measures: fractionated administration to reduce hematological toxicity and co-infusion with positively charged amino acids to reduce late nephrotoxicity. Overall, the clinical results are promising, but few patients are cured and the main benefit is prolonged survival, symptom relief and improvement of quality of life^6^. The EMA and FDA-approved clinical protocols only allow for up to four treatment cycles of 7.4 GBq ^177^Lu-octreotate. Thus, it is imperative to develop better and optimized therapeutic options for NETs. By co-treating with radioprotective drugs, normal tissue toxicity may be reduced, and higher ^177^Lu activity levels can possibly be administered, which could result in increased anti-tumor effect and cure rate. Furthermore, patients currently excluded to MRT due to impaired renal function might also be treated.

A recombinant form of the human antioxidant α_1_-microglobulin (A1M) has been suggested as a possible kidney protective agent during molecular radionuclide therapy (MRT)^7–10^. A1M is a 26 kDa plasma and tissue protein that protects human tissues against oxidative damage by radical scavenging, and acting as a reductase and inhibitor of oxidation^11^. A1M has been shown to co-localize with radiolabeled somatostatin analogues in the mouse kidney cortex and share similar biodistribution and pharmacokinetics^7,8^. Furthermore, A1M significantly suppressed radiobiological effects in the renal cortex through reduced expression of apoptosis and stress related genes for ^177^Lu-octreotate treated mice^10^.

Apoptosis is triggered when a cell suffers a fatal injury that is too error-prone to be repaired, and is assumed to be a major cell death mechanism after ionizing radiation exposure^12^. Apoptosis is understood to be initiated either via the intrinsic or extrinsic pathways. The former is triggered by intracellular stress and the latter by death ligands from neighboring cells, signaling via membrane-bound death receptors such as FAS or TNF. Regardless of the initiation, the routes converge at the mitochondria by releasing apoptotic substrates that start a downstream cascade of caspase activation leading to the execution of apoptosis^12^. The BCL2 family strongly regulates the mitochondrial membrane permeabilization and consists of proteins with pro and anti-survival influence, such as BAX and BCL2.

The purpose of using radioprotectors in MRT is to reduce normal tissue radiotoxicity and thereby allow a more aggressive treatment to improve the therapeutic outcome. One of the main challenges is ensuring that, while normal tissues are to some extent protected against radiation effects, tumor tissue remain unprotected. We recently showed that combining ^177^Lu-octreotate with A1M did not negatively affect the therapeutic response of ^177^Lu-octreotate in a human NET (GOT1) mouse model^9^. There is, however, no clear understanding of the biological mechanisms underlying these findings yet.

The aim of this work was to evaluate the apoptotic response in patient-derived GOT1 NE tumors in mice 1 and 7 days after administration of ^177^Lu-octreotate, ^177^Lu-octreotate combined with A1M, or A1M alone.

## Materials and methods

### ^177^Lu-octreotate

^177^Lu-octreotate (Nuclear Research and Consultancy Group, IDB Holland) was prepared according to the manufacturer’s instructions. The amount of peptide bound ^177^Lu (> 99%) was measured by instant thin layer chromatography (ITLC), using Whatman Chromatography paper (3 mm, GE Healthcare UK Limited, Amersham, England) and 0.1 M sodium citrate (Labservice AB, Sundsvall, Sweden). Syringes containing 30 MBq ^177^Lu-octreotate (in 0.1 ml) were prepared and measured with a well-type ionization chamber (CRC-15R; Capintec, New Jersey, USA). Administered activity to each animal was determined from the ^177^Lu activity in syringe before and after injection.

### Recombinant A1M

Human recombinant A1M (RMC-035), henceforth referred to as A1M and dilution solution containing sterile endotoxin-free 10 mM Na_3_PO_4_ (pH 7.4), 0.15 M NaCl, and 12 mM histidine were supplied by A1M Pharma AB (Lund, Sweden). A1M was diluted from a concentration of 5.9 mg/ml to a concentration of 0.77–1.2 mg/ml and administered at 5.0 mg/kg body weight, determined at the time of injection.

### Tumor model

The GOT1 cell line was derived from a small-intestine NET collected from a patient at surgery in the 1990ies^13,14^. Since GOT1 is slowly growing, the construction of the GOT1 tumor nude mouse model starts with the first generation of young (4–6 weeks old) female nude mice subcutaneously injected with GOT1 cells. When larger solid tumors have been developed, they are extracted and divided into ca 1 mm^3^ GOT1 tissue samples that are subcutaneously transplanted on new young female nude mice under anesthesia by i.p. injection of Ketaminol^®^ vet. (Intervet AB, Stockholm, Sweden) and Domitor vet. (Orion Pharma AB Animal Health, Sollentuna, Sweden). An i.p. injection of Antisedan vet. (Orion Pharma AB Animal Health) was used as antidote. By further serial transplantation of tumor tissue, several mouse generations can be derived and used for experimental studies. For a successful model, tumors should have similar characteristics to those seen in patients, such as preserved growth characteristics, receptor expression, and radiolabeled somatostatin analogue uptake. These properties are regularly checked. Since there is some reduction of receptor expression with each generation in vivo, especially regarding SSTRs, new first generation tumor-bearing mice are routinely created from GOT1 cells. With this approach, many studies have been performed during this extended time.

In the current study, female nude mice (Janvier, France and Charles River, Germany) in a later generation had subcutaneous GOT1 tumors. The animals were kept under a standard laboratory day and night cycle and were given water and food ad libitum. All animal procedures were approved by the Ethics Committee for Animal Research in Gothenburg, Sweden (approval 107-2015), and carried out in accordance with relevant guidelines and regulations and are reported following ARRIVE guidelines.

### Animal experiment

The animals were divided into four groups. Three groups received a tail vein injection of either ^177^Lu-octreotate (30 MBq, n = 6), ^177^Lu-octreotate + A1M (30 MBq, 5 mg/kg, n = 6) or A1M only (5 mg/kg, n = 6). In addition, a control group was sham treated with saline solution (n = 4). Cardiac puncture under anesthesia with Pentobarbitalnatrium vet. (Apotek Produktion & Laboratorier AB, Huddinge, Sweden) was used to terminate half of the number of animals in each group at day 1 and the remaining animals at day 7. Digital calipers were used to measure tumor volume on the day before injection and on the day of termination. To estimate the tumor volume, we assumed an ellipsoid shape

$$V = \frac{{\pi \cdot a \cdot b \cdot c}}{6}$$

where a is the longest diameter and b and c are perpendicular diameters. Mean tumor volume at study start was 0.95 ml (SEM 0.08 ml), 0.83 (SEM 0.14 ml), 1.6 ml (SEM 0.5 ml) and 0.45 ml (SEM 0.06 ml) in the ^177^Lu-octreotate, ^177^Lu-octreotate + A1M, A1M only and control group, respectively. A non-curative ^177^Lu activity level was chosen to avoid complete tumor regression. After termination, a part of each tumor was flash frozen in liquid nitrogen and stored at − 80 °C, while the other part was fixed in formalin.

### Absorbed dose to tumor

The absorbed dose, D, to the tumor in mice injected with ^177^Lu-octreotate was calculated according to the MIRD pamphlet 21 formalism^15^

$$D = \frac{{\tilde{A}}\Delta \phi }{m},$$

where Ã is the time-integrated activity in tumor, $\Delta$ is the total electron energy emitted by the radionuclide per disintegration (147.9 keV for ^177^Lu)^4^, $\phi$ is the absorbed fraction of energy from the emitted particles, and *m* is the tumor mass. At each time point, the activity in tumor tissue was calculated using data of activity concentration from a previous biodistribution study of ^177^Lu-octreotate in GOT1-bearing nude mice^16^. Ã was approximated using a trapezoid function with the initial activity of zero, A(t = 0) = 0. $\phi$ was set to 1, assuming local energy absorption and neglecting energy deposition of photons^5,17^.

### Gene expression analysis

Total RNA was extracted from frozen tumor tissue samples according to the manufacturer’s protocol using the RNeasy Lipid Tissue Mini Kit (QIAGEN, Valencia, USA). The purity, quality and concentration of isolated RNA were assessed with Nanodrop 1000 Spectrometer (Thermo Scientific), RNA 6000 Nano LabChip Kit and Agilent 2100 Bioanalyzer (both from Agilent Technologies) (RIN > 7), and Qubit 3.0 Fluorometer (Thermo Fisher Scientific) (> 40 μg/ml), respectively. RNA was transcribed into cDNA using the RT^2^ First Strand Kit (QIAGEN, Valencia, USA). The synthesized cDNA was analyzed with RT^2^ PCR profiler arrays using RT^2^ SYBR Green Mastermix (QIAGEN, Valencia, USA), according to the manufacturer’s instructions. Each sample was analyzed with one array, specified for human apoptosis (PAHS-012ZF, QIAGEN) and measured using Roche LightCycler^®^ 480 system (QIAGEN) at TATAA Biocenter (Gothenburg, Sweden). The profiling array consists of 96 wells with primers for 84 genes of interest and five housekeeping genes (*ACT*, *B2M*, *GAPDH*, *HPRT1*, and *RPLP0*). Of the 84 genes, 51 were categorized as pro-apoptotic, 25 as anti-apoptotic and eight were associated with apoptosis regulation with both pro- and anti-apoptotic functions, partly based on the categorization by the manufacturer, but also including information from GeneCards (https://www.genecards.org). The remaining wells were used to measure genomic DNA contamination, positive PCR, and reverse transcription controls. All genes were normalized against the geometric mean of all housekeeping genes included in the array. Regulated fold change (FC) was calculated relative to the control group using the 2^–∆∆Ct^ method with a cut-off value of |FC|≥ 1.5. All figures were produced in GraphPad Prism version 9.3.1 for macOS (https://www.graphpad.com/scientific-software/prism/).

### Immunohistochemistry

Formalin-fixed paraffin-embedded (FFPE) GOT1 tumor samples were sectioned into 4um slices and dried for 1 h at 60° before deparaffinization and antigen retrieval with EnVision Flex target retrieval solution at pH 9 using DAKO PTLink (Agilent Technologies, Santa Clara, California, USA). Immunohistochemical staining was performed for FAS, survivin (BIRC5), BIRC3 (cIAP2), and TNFRSF10B (to visualize expression of these proteins), cleaved CASP3 and annexin V (as general apoptosis markers), and SSTR2 and chromogranin A (CHGA) (as NET markers). As positive control for SSTR2 and CHGA, a human small intestine NET was used.

Immunohistochemical staining was carried out using DAKO Autostainer Plus with EnVision peroxidase blocking reagents (both from Agilent Technologies, Santa Clara, California, USA), followed by staining with the primary antibodies (Table 1). Next, FLEX/HRP was applied, and the tissues were stained with DAB (3,3’-diaminobenzidine) and counterstained with EnVision FLEX hematoxylin (all from Agilent Technologies, Santa Clara, California, USA). Between each staining step, sections were washed with EnVision FLEX wash buffer (1x) (Agilent Technologies, Santa Clara, California, USA) and subsequently rinsed with deionized water. Sections were then dehydrated in ethanol (75, 95 and 99%) and cleared twice in xylene before coverslips were mounted with Pertex mounting medium for light microscopy (Histolab Products AB, Askim, Sweden). The stained tumor sections were digitalized using a Leica SCN400 at 40×. The pathologist (AK) evaluated each staining and estimated the percentage of positively stained cells and the intensity of the staining. A histological score (H-score) was determined according to the staining intensity (weak = 1, moderate = 2, strong = 3), which was then multiplied by the percentage of positively stained cells, and the three termes summed to give the H-score value ranging from 0 to 300.

**Table 1:** Primary antibodies used for immunohistochemical staining of FFPE GOT1 tumor sections.

Origin	Antibody (antigen)	Dilution	Cat. no,	Company
Rabbit monoclonal	Anti-Fas (FAS)	1:250	ab133619	Abcam, Cambridge, UK
	Anti-survivin (survivin)	1:250	ab76425	Abcam, Cambridge, UK
	Anti-cIAP2 (BIRC3)	1:300	ab32059,	Abcam, Cambridge, UK
	Anti-Chromogranin A (CHGA)	1:50	ab68271	Abcam, Cambridge, UK
	Anti-SSTR2 (SSTR2)	1:600	ab68271	Abcam, Cambridge, UK
	TRAIL-R2 (TNFRSF10B)	1:100	MA-532693	Invitrogen, Waltham, Mass, USA
	Cleaved-Casp3 (CASP3)	1:50	#966	Cell Signaling Technology, Danvers, Mass, USA
Mouse monoclonal	Anti-annexin V (annexin V)	1:50	ab54775	Abcam, Cambridge, UK

The origin, name, dilution factor used, and manufacturers of the antibodies are listed.

### Apoptotic index

Apoptotic index is defined as the percentage of cells that are in apoptosis. For histological analyses of tumor samples, FFPE GOT1 were deparaffinized and rehydrated followed by staining with hematoxylin and eosin (H&E). From each H&E stained tumor section, the pathologist (AK) selected three regions of interest (ROIs) representing low, moderate, and high levels of apoptosis cells. All ROIs contained at least 1000 cells. An automated count of all the cells within each ROI was performed using QuPath version 0.4.1 (https://qupath.github.io/), an open-source software program for digital pathology analysis^18^. Then, apoptotic cells were identified and counted manually in each ROI based on the presence of fragmented nuclei and eosin-rich cytoplasm. The apoptotic index for each tumor was determined by averaging the percentage of apoptotic cells in the three ROIs per tumor section.

### Statistical analysis

The gene expression levels (treated vs control) were analyzed by Welch’s t-test, and p < 0.05 was considered statistically significant. Kruskal-Wallis one-way ANOVA followed by Welch’s t-test was used to determine statistically significant differences between the treated groups for the gene with statistically significant expression in at least one of the groups (p < 0.05). Apoptotic index was analyzed using one-way ANOVA followed by Welch’s t-test. The statistical analysis of expression data and apoptotic index was performed using Perseus software version 1.6.15.0 (https://maxquant.net/perseus/)^19^. GraphPad Prism version 9.3.1 (https://www.graphpad.com/scientific-software/prism/) was used to analyze the differences in H-scores between antibody stainings using one-way ANOVA followed by Tukey's posthoc test.

### Gene ontology analysis and reactome pathway analysis

Differentially expressed genes were used to determine biological processes associated with cell death using the Gene ontology (GO) database^20^ (http://amigo.geneontology.org/amigo). Pathway analysis was performed using the Reactome Pathway Database^21^ (https://reactome.org/) to identify the specific pathways in which the significantly expressed genes are involved.

## Results

### Tumor volume and absorbed dose estimation

Tumor volume decreased by 47% (SEM 4%) and 35% (SEM 3%) during 7 days after administration of ^177^Lu-octreotate and ^177^Lu-octreotate combined with A1M, respectively, from their initial values. For the control and A1M only groups, tumor volume increased by 53% (SEM 3%) and 48% (SEM 7%), respectively, during 7 days. After 1 day after injection, the mean absorbed dose to tumor tissue was 0.99 Gy (SEM 0.01 Gy) in the ^177^Lu-octreotate group and 0.92 Gy (SEM 0.03 Gy) in the ^177^Lu-octreotate + A1M group. The corresponding mean absorbed dose at 7 days after injection was 5.4 Gy (SEM 0.2 Gy) and 5.6 Gy (SEM 0.3 Gy) in the ^177^Lu-octreotate group and ^177^Lu-octreotate + A1M group, respectively.

### Gene expression

**Figure 1:**
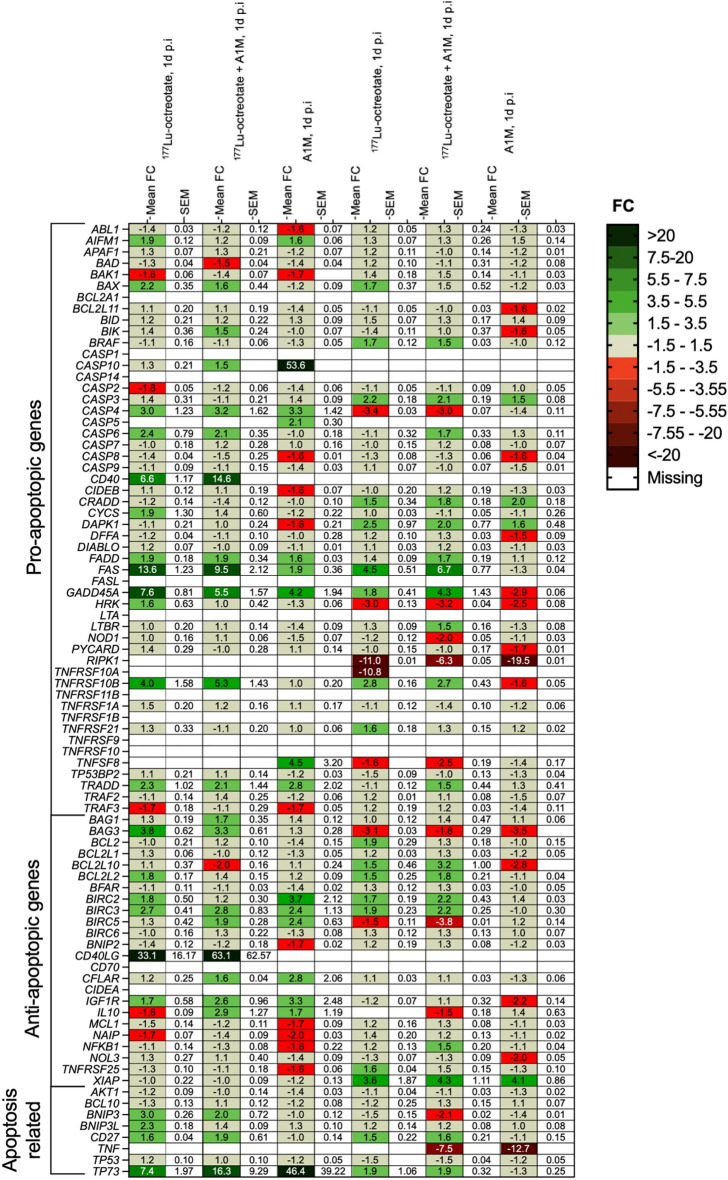
mRNA expression of apoptosis related genes in GOT1 tumor tissue of mice injected with ^177^Lu-octreotate, ^177^Lu-octreotate + A1M, or A1M collected at 1 and 7 days after treatment (left to right). Gene regulation is expressed as fold change relative to the control group (FC) and reported as mean and SEM. Three and two animals with GOT1 tumors are included in each treatment group and controls, respectively, at both 1 and 7 days after study start. Red and green shades represent down- and upregulation, respectively, with more than 1.5-fold expression. Grey color indicates regulation less than 1.5-fold expression. Missing data in all tumors is represented by white color. Statistical analysis was performed using Perseus software 1.6.15.0 (https://maxquant.net/perseus/) and the figure was produced using GraphPad Prism version 9.3.1 (https://www.graphpad.com/scientific-software/prism/).

Figure 1 shows the expression levels of apoptosis involved genes in GOT1 collected at 1 and 7 days after administration of either ^177^Lu-octreotate, ^177^Lu-octreotate + A1M or A1M. Totally, 73 of the 84 studied genes were detectable in at least one of the groups after 1 or 7 days. In general, gene expression patterns were similar between the ^177^Lu-octreotate and ^177^Lu-octreotate + A1M groups at each time-point, with some exceptions. In these groups, high upregulation was found for the pro-apoptotic genes *CD40*, *FAS* and *GADD45A*, for the anti-apoptotic *CD40LG* gene, and for the apoptosis-related *TP73* gene on day 1. On day 7, pro-apoptotic genes *RIP1K* and *TNFRSF10A* and the apoptosis-related gene *TNF* were all down-regulated. The general expression pattern differed between the A1M group and the irradiated groups at each time-point. For the A1M group, high regulation (up) was only noted for pro-apoptotic gene *CASP10* and the apoptosis-related *TP73* gene after 1 day, while the pro-apoptotic gene *RIP1K* and the apoptosis-related gene *TNF* were both down-regulated after 7 days.

#### Statistically significant regulated genes

**Figure 2:**
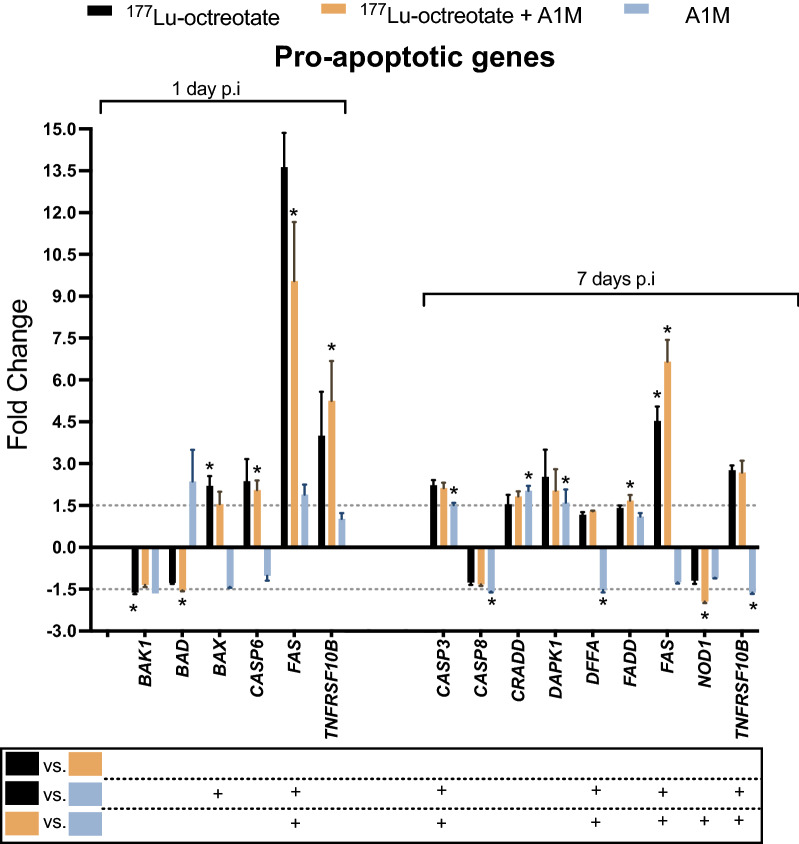
Pro-apoptotic response in GOT1 tumors after exposure to ^177^Lu-octreotate, ^177^Lu-octreotate + A1M, and A1M alone. The figure shows expression of all pro-apoptotic genes with significantly different expression compared to controls (p < 0.05 and regulated |FC|≥ 1.5) in at least one of the three groups. Dashed lines represent |FC|= 1.5, and the box beneath the graph shows results from group comparisons from one-way ANOVA tests, where “+” represents a statistically significant difference (p < 0.05) between groups. Tumors from three and two mice were used in the treatment and control group, respectively, with an exception for *DAPK1* in A1M only group (n = 2). Error bars represent SEM. *p < 0.05 (0.0010–0.050). Statistical analysis was performed using Perseus software 1.6.15.0 (https://maxquant.net/perseus/) and the figure was produced using GraphPad Prism version 9.3.1 (https://www.graphpad.com/scientific-software/prism/).

**Figure 3:**
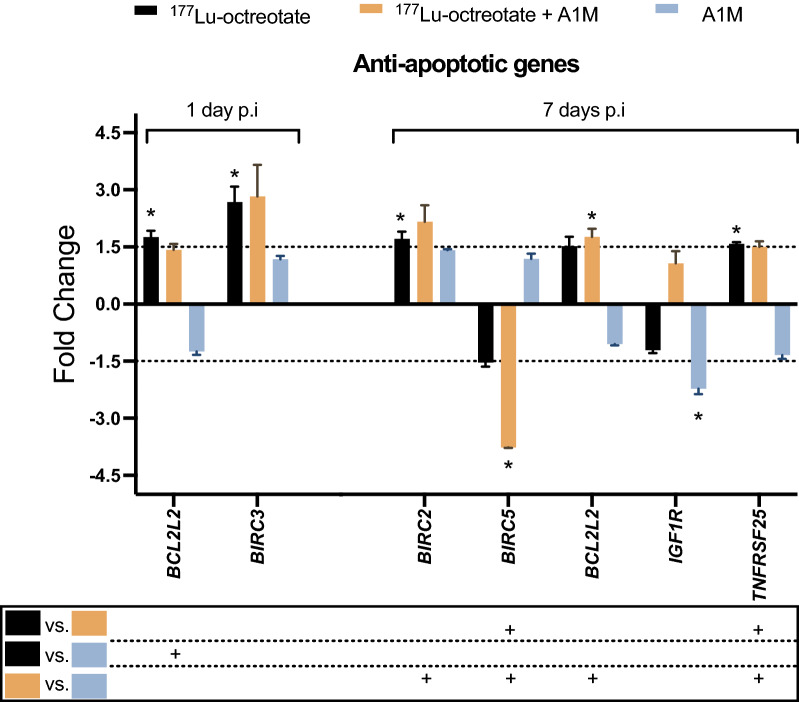
Anti-apoptotic response in GOT1 tumors after exposure to ^177^Lu-octreotate, ^177^Lu-octreotate + A1M, and A1M alone. The figure shows **e**xpression of all anti-apoptotis related genes with significantly different expression compared to controls (p < 0.05 and regulated |FC|≥ 1.5) in at least one of the three groups. *BIRC3* and *BIRC5* showed highest regulation. Dashed lines represent |FC|= 1.5, and the box beneath graphs shows results from group comparisons with one-way ANOVA tests, where “+” represents a statistically significant difference (p < 0.05) between groups. Tumors from three and two mice were used in the treatment and control group, respectively. Error bars represent SEM. *p < 0.05 (0.017–0.048). Statistical analysis was performed using Perseus software 1.6.15.0 (https://maxquant.net/perseus/) and the figure was produced using GraphPad Prism version 9.3.1 (https://www.graphpad.com/scientific-software/prism/).

Gene expression of statistically significant up- or downregulated genes compared to controls in at least one of the three groups are shown in Figs. 2 and 3. The total number of genes with |FC|≥ 1.5 and statistically significant regulation compared to controls (p < 0.05) was eight on day 1 and 14 on day 7. Only genes classified as either pro- or anti-apoptotic were statistically significant regulated, not any of the genes categorized as apoptosis-related (with both pro- and anti-apoptotic functions).

1 day after injection, at least one group showed statistically significant changes in *BAK1, BAD, BAX, CASP6, FAS*, and *TNFRSF10B* expression (Fig. 2). In the ^177^Lu-octreotate group, *BAK1* (down-regulated) and *BAX* (upregulated) were detected. The combination group showed differential expression of *BAD, CASP6, FAS*, and *TNFRSF10B*, with *BAD* downregulated and the other genes upregulated. The highest expression levels were found for *FAS* and *TNFRSF10B*. In the A1M group, no statistically significant regulated pro-apoptotic genes compared to controls were detected after 1 day.

After 1 day, the anti-apoptotic genes *BCL2LC* and *BIRC3* were significantly upregulated in ^177^Lu-octreotate group, while neither the A1M group nor the combination with ^177^Lu-octreotate significantly altered the expression of anti-apoptotic genes (Fig. 3).

7 days after injection, the expression of nine pro-apoptotic genes was statistically significant changed compared to sham controls, in at least one group: *CASP3, CASP8, CRADD, DAPK1, DFFA, FADD*, *FAS, NOD1* and *TNFRSF10B* (Fig. 2). *FAS* was upregulated in the ^177^Lu-octreotate and ^177^Lu-octreotate + A1M groups. In the combination group, also *FADD* was upregulated, and *NOD1* downregulated. The highest expression levels of pro-apoptotic genes were found for *FAS*. There were no statistically significant differences in the expression pattern of pro-apoptotic genes between the ^177^Lu-octreotate and combination groups. In the A1M group, *CASP3, CRADD,* and *DAPK1* were statistically significant upregulated, and *CASP8, DFFA* and *TNFRSF10B* downregulated after 7 days.

On day 7, the expression of five anti-apoptotic genes were significantly altered in at least one of the groups: *BIRC2*, *BIRC5*, *BCL2L*, *IGF1R* and *TNFRSF25* (Fig. 3). None of these genes were in common for the groups. In the ^177^Lu-octreotate group, the expression of *BIRC2* and *TNFRSF25* was statistically significant upregulated. In the combination group, the *BIRC5* and *BCL2L2* genes were down- and upregulated, respectively. 7 days after administration of A1M alone, only *IGFR1* was found to be differentially expressed (downregulated).

#### Highly regulated genes

The expression of genes with mean |FC|> 7 in at least one group that were not statistically significantly changed compared to sham controls are listed in Table 2. Reasons for not being statistically significant different were a large range of FC values or too few data (further discussed below).

**Table 2:** Genes with high FC > 7 in at least one group at 1 and 7 days after injection that were not statistically significant expressed compared to corresponding controls.

Time (days)	Gene	^177^Lu-octreotate		^177^Lu-octreotate + A1M		A1M	
		FC median (range)	n, p	FC median (range)	n, p	FC median (range)	n, p
1	*CASP10*, pro	1.3 (1.1–1.5)	n = 2, p = 1	1.5	n = 1, p = 1	54	n = 1, p = 1
	*CD40*, pro	6.6 (5.5–7.8)	n = 2, p = 1	15	n = 1, p = 1	–	n = 0
	*GADD45A*, pro	7.7 (6.1–8.9)	n = 3, p = 0.1	4.7 (3.4–8.6)	n = 3, p = 0.07	3.9 (1.1–7.7)	n = 3, p = 0.2
	*CD40LG*, anti	40 (2.4–57)	n = 3, p = 1	63 (0.5–130)	n = 2, p = 1	–	n = 0
	*TP73,* apop-rel	5.9 (5.0–11)	n = 3, p = 0.26	11 (3.7–34)	n = 3, p = 0.16	9.1 (5.3–125)	n = 3, p = 0.12
7	*RIPK1,* pro	−10 (−10 to −13)	n = 3, p = 1	−6.8(−4.8 to −8.8)	n = 2, p = 1	−20 (−17 to −23)	n = 2, p = 1
	*TNFRSF10A,* pro	−11	n = 1, p = 1	–	n = 0	–	n = 0
	*TNF,* apop-rel	–	n = 0	−7.5	n = 1, p = 1	−13	n = 1, p = 1

Expression is given as median FC (range), n is the number of data points, and the p-value is given from statistical analysis versus corresponding controls. Pro, anti and apop-rel are abbreviations of pro-apoptotic, anti-apoptotic and apoptosis-related, respectively. Statistical analyses were performed using Perseus software 1.6.15.0 (https://maxquant.net/perseus/).

After 1 day, high expression was observed for the pro-apoptotic *CD40* and *GADD45A* genes, upregulated in both groups that received ^177^Lu-octreotate. One animal in the A1M group showed upregulation of *CASP10*, whereas no data were obtained for *CASP10* for the remaining animals in the group. The anti-apoptotic gene *CD40LG* was upregulated in both groups that received ^177^Lu-octreotate. High expression was also observed for the apoptosis-related *TP73* gene, which was upregulated in all groups.

After 7 days, the pro-apoptotic genes *RIPK1* and *TNFRSF10A* were down-regulated in all groups and in the ^177^Lu-octreotate group, respectively (data missing from *TNFRSF10* for the other groups). The apoptosis-related TNF gene was down-regulated in both groups that received A1M, but data is missing for the ^177^Lu-octreotate group.

### Histological analyses

**Figure 4:**
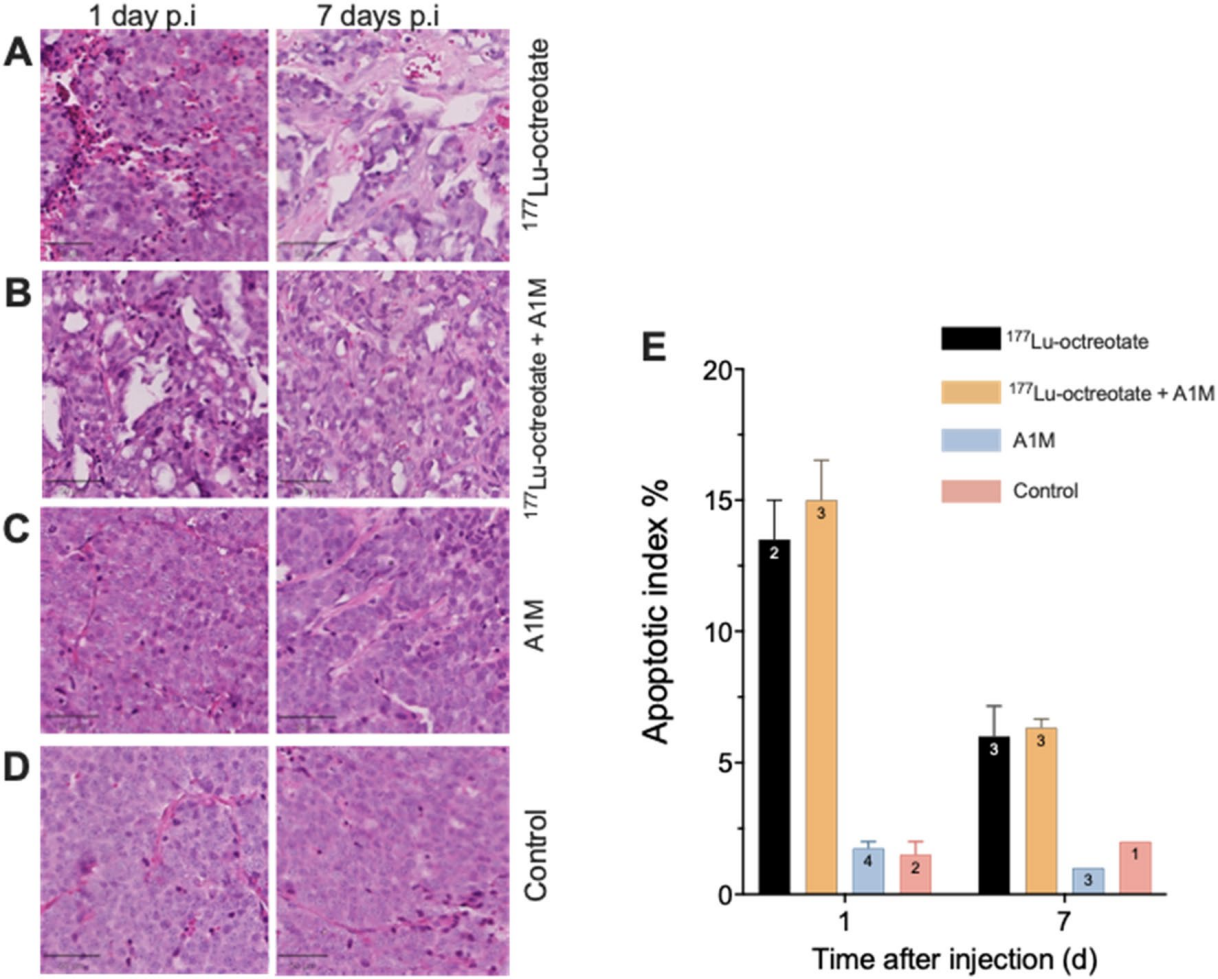
Sections of GOT1 tumors stained with H&E 1 and 7 days after injection of (**A**) ^177^Lu-octreotate, (**B**) ^177^Lu-octreotate + A1M, or (**C**) A1M, and (**D**) sham-treated controls. Black bar in bottom left corner corresponds to 20 µm. (**E**) Mean apoptotic index for each group at 1 and 7 days. Error bars represent SEM and sample size is written in the bars. Figure was produced using GraphPad Prism version 9.3.1 (https://www.graphpad.com/scientific-software/prism/).

Figure 4A–D shows typical GOT1 tumor sections stained with H&E and Fig. 4E illustrates the apoptotic index in GOT1 tumors from the different groups 1 and 7 days after injection. Injection of ^177^Lu-octreotate or ^177^Lu-octreotate + A1M resulted in small necrotic foci with inflammatory infiltrates 1 day after injection and apoptotic index were significantly higher in ^177^Lu-octreotate group and ^177^Lu-octreotate + A1M group compared to control. There was no significant histological difference between the A1M group and control at day 1.

On day 7, irradiated tumors from both groups given ^177^Lu-octreotate showed necrosis in their center and fibrosis. The apoptotic index was reduced on day 7 for irradiated tumors, irrespective of A1M co-administration compared with day 1. In tumors from the control group and the A1M group, no significant structural changes or tissue damage were observed. The unirradiated tumors contained a small proportion of apoptotic cells, but also mitotic cells, which were not observed to the same degree in the irradiated tumors.

The apoptotic index was determined for GOT1 tumors from the different groups 1 and 7 days after injection (Fig. 4E). Statistically significant differences in apoptotic index on day 1 were found between ^177^Lu-octreotate + A1M and A1M groups (p = 0.01), and ^177^Lu-octreotate + A1M and controls (p = 0.008). After 7 days, corresponding differences were shown between ^177^Lu-octreotate and A1M groups (p = 0.049), and between ^177^Lu-octreotate + A1M and A1M groups (p = 0.004).

**Figure 5:**
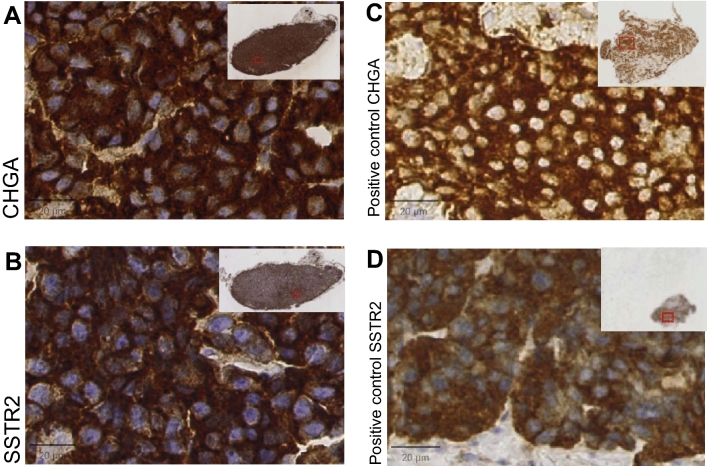
Expression of CHGA and SSTR2 in representative GOT1 tumors (**A,B**) and human small intestinal NETs used as positive controls (**C,D**). The two NET markers SSTR2 and CHGA were highly expressed in all GOT1 tumors included in the study. Black bar = 20 μm. Figure was produced using GraphPad Prism version 9.3.1 (https://www.graphpad.com/scientific-software/prism/).

All GOT1 tumors stained positive for the NET markers CHGA and SSTR2 (Fig. 5). Results from staining against the apoptotic markers are shown in Supplementary Figs. 1–5. H-score values for the different markers are found in Supplementary Fig. 6. 1 day after ^177^Lu-octreotate injection, with and without A1M, GOT1 tumors stained against annexin V showed predominant membrane and cytoplasmic staining (Supplementary Fig. 1). However, on day 7, most positive staining was cytoplasmic in both groups. In the A1M group and the control group, most of the positive staining was cytoplasmic, while two tumors in each group showed some membrane staining. Regarding survivin, a low percentage of strong staining, mainly nuclear, was found in GOT1 tumor from the ^177^Lu-octreotate and ^177^Lu-octreotate + A1M groups on day 1 (Supplementary Fig. 2). After 7 days, strong positive staining was found in the ^177^Lu-octreotate group, whereas weak to moderate staining was found in the combination group. Also, the percentage of positively stained cells was low in both ^177^Lu-octreotate irradiated groups compared to the A1M and the control groups, which showed a higher percentage of stained cells, mainly strong nuclear staining which did not change significantly over time. All GOT1 tumor sections, irrespective of treatment, contained weak cytoplasmic staining of TNFRSF10B (Supplementary Fig. 3). After 1 day, the staining against FAS (Supplementary Fig. 4) was found predominantly in the cytoplasm for tumors from the^177^Lu-octreotate + A1M group, with intensities varying between weak and strong, and a similar trend was seen after 7 days. Tumors from the ^177^Lu-octreotate group did not show any positive staining after 1 day, while after 7 days, the staining was weak to moderate in cytoplasm. Tumors from the A1M and control groups showed a low percentage of weak to moderate positive staining against FAS after 1 day, but a low percentage of cells were positively stained. After 7 days, a higher percentage of cells were stained with a similar intensity. Stainings against cleaved CASP3 in all GOT1 tumors were found to be cytoplasmic (Supplementary Fig. 5). After 1 day, weak staining with moderate intensities and weak to strong staining against cleaved CASP3 were found in tumors from the ^177^Lu-octreotate and combination group, respectively, although a higher percentage of cells were positive in the ^177^Lu-octreotate group. A similar trend for cleaved CASP3 was observed with time. In the A1M group, a low percentage of cells were positively stained with varying intensity. Regarding the control group, strong to weak staining against cleaved CASP3 was observed and persisted over time.

### Gene Ontology and pathway analysis

GO terms of biological processes related to cell death annotated to the differentially expressed genes found in this study are shown in Supplementary Table S1. GO analysis revealed that pro-apoptotic genes were generally annotated with more GO terms related to cell death than anti-apoptotic ones. Except for programmed cell death, pathway analysis showed that the ^177^Lu-octreotate group had more immunological pathways involved, whereas the ^177^Lu-octreotate + A1M group had more pathwats related to metabolism and disease response on day 1. Similar trends were observed for the irradiated groups at day 7.

## Discussion

Optimized protection of kidneys during ^177^Lu-octreotate treatment of NET may allow administration of further treatment cycles, resulting in higher amount of ^177^Lu-octreotate administered, higher absorbed dose to tumor and hopefully higher cure rate. The endogenous antioxidant A1M has recently been suggested as a kidney radioprotector and results regarding normal tissue protection are promising in animal studies^7,8,22^. Importantly, it is also necessary to assess its influence on malignant tissue, to ensure that A1M does not also protect the tumor tissue. Hitherto, the therapeutic effect of co-treatment with A1M in MRT has only been studied in tumor models by evaluating the change in tumor volume^9,23^. It is possible that the radiobiological effects can differ substantially between tumor and normal tissues, depending on genetic alterations in the malignant tissue.

According to a previous study, the addition of the antioxidant *N*-acetylcysteine or vitamin E resulted in faster tumor progression, increased number of tumors in mice with B-RAF- or K-RAS-induced lung cancer^24^. Furthermore, exposure of mouse and human lung cancer cells to these antioxidants reduced expression of the tumor suppressor gene p53, reduced DNA damage and reduced production of reactive oxygen species (ROS), resulting in increased cell proliferation^24,25^. These results clearly demonstrate the importance of investigating the potential proliferative effects of antioxidants on tumor tissue, and also potential radioprotective effects of antioxidants. We therefore conducted the present study to investigate the response in GOT1 tumor tissue when exposed to ^177^Lu-octreotate with or without A1M. To gain a deeper understanding of the transcriptional effects of radiation, we focused on the apoptotic response induced by radiation response.

Overall, few of the studied genes were up- or down regulated in the present study, indicating that ionizing radiation induces apoptosis by selected apoptotic processes, not involving a general gene response. After ^177^Lu-octreotate irradiation, with or without A1M co-administration, the response at both 1 and 7 days displayed similar pattern in GOT1 tumors, regarding both responding genes, although not always statistically significant, and the regulation pattern, both in direction and fold change. Only genes categorized as either pro- or anti-apoptotic were among the statistically significant regulated ones.

Most of the pro-apoptotic genes were upregulated, and few were downregulated at both time points, both for the statistically significantly altered ones and overall. Two pro-apoptotic genes were upregulated at both time-points in the irradiated tumors: *FAS* (Fas cell surface death receptor) and *TNFSF10B* (Tumor necrosis factor receptor superfamily, member 10b)*,* both demonstrating the highest mean gene regulation*.* These genes are both coding for members of the tumor necrosis factor (TNF) superfamily and so-called death receptors^26^, indicating that ^177^Lu-octerotate may induce apoptosis via the extrinsic pathway.

Two pro-apoptotic genes were observed to be downregulated at day 1, *BAD* (*BCL2 associated agonist of cell death*) and *BAK1 (BCL2 antagonist/killer 1)*, which both are members of the B-cell lymphoma 2 (Bcl2) family^26^. These genes encode for proteins that stimulate the breakdown of mitochondrial membrane, a key event in apoptosis, and are inhibited by high abundance of anti-apoptotic members of the Bcl2 family of proteins, such as BCL2^27^. In this study, both *BAK1* and *BAD* were downregulated in both irradiated groups (regardless of A1M administration). Ionizing radiation may induce genotoxic stress and beside cell death, other survival pathways inhibiting apoptosis may be inducted simultaneously resulting in suppression of some pro-apoptotic proteins or increase in anti-apoptotic activity. Another member of the Bcl-2 family, *BAX* (BCL2 associated X, apoptosis regulator), encodes for an apoptosis activator that is important in radiation sensitivity and radiation-induced cell death^26,27^. After 1 day, *BAX* expression was increased in both irradiated groups regardless of A1M administration, although not statistically significant in the combination group. Similar findings were also observed for the expression of the *caspase 6* (*CASP6)* apoptosis executioner gene, where radiation exposure with and without A1M led to upregulation.

Among the anti-apoptotic genes that were detectable with more than 1.5-fold expression, most were upregulated (Fig. 1). Two of the genes with anti-apoptotic activity were significantly upregulated after 1 day in both groups treated with ^177^Lu-octreotate, the *BCL2-like 2* (*BCL2LC)* and baculoviral IAP repeat containing 3 (*BIRC3)* genes. Furthermore, the *BCL2LC* gene was upregulated after 7 days after irradiation with and without A1M, together with the baculoviral IAP repeat containing 2 (*BIRC2)* and TNF receptor superfamily member 25 (*TNFRSF25)* genes. Inhibitors of Apoptosis proteins (IAPs) act mainly to reduce caspase activity and, therefore, hinder execution of apoptosis^28^. IAPs included in the panel of investigated genes were *BIRC2,3,5, XIAP* and *NIAP*. In this study, radiation-induced overexpression of *BIRC2* and *BIRC3* was observed at 1 and 7 days. The expression of *BIRC2* and *BIRC3* was in the same direction in the ^177^Lu-octreotate as in the combination group. However, at 7 days, a stronger downregulation of *BIRC5* was observed in the combination group compared to ^177^Lu-octreotate monotherapy, suggesting that A1M may enhance the effect of ^177^Lu-octreotate exposure. It has been reported that *BIRC5* is commonly overexpressed in gastrointestinal NETs and other cancer types, and overexpression has been related to low radiosensitivity and worse prognosis^29–31^.

After irradiation, tumor sections showed higher apoptotic index compared to unirradiated controls, but there was no statistically significant difference between ^177^Lu-octreotate and ^177^Lu-octreotate + A1M groups, suggesting that the antioxidant did not alter radiation-induced apoptosis. In agreement with previous studies on GOT1 tumor tissue, the apoptotic index decreased from day 1 to day 7^32^. At 7 days, a more pronounced degree of necrosis and onset of fibrosis were observed than on day 1. Moreover, both irradiated groups, with and without A1M, showed a reduction in tumor volume over time, which was not observed in the controls and A1M alone groups. We have previously shown that GOT1 tumor growth after injection with A1M follows a similar pattern as untreated controls over time^9^. Since the apoptotic index in the A1M group and controls was similar, it may be concluded that A1M does not alter the apoptotic response in the short term. However, further research is needed to establish this finding and to determine whether A1M has any long-term effects on tumor tissue.

Summarizing all these data, co-injection with A1M did not negatively influence the effects of ^177^Lu-octreotate on GOT1 tumor tissue at any of the studied time-points. These findings are in agreement with our previous morphological studies on GOT1 tumors in mice injected with 30 MBq ^177^Lu-octreotate that showed a high frequency of apoptotic cells at 1 and 3 days, but not 7 days after administration^32^. A late pro-survival response at day 7 might be anticipated in the remaining tissue.

It is also possible that the protective effect of A1M can differ between tissue types after ^177^Lu-octerotate exposure. In the present study on GOT1 tumors, co-administration of A1M showed, for example, no effect on the radiation induced *FAS* and *TNFRSF10B* expression*.* Kristiansson et al.^10^ showed that co-administration of A1M with ^177^Lu-octreotate reduced the expression of *TNFRS10B* in mouse kidneys and suggested this result as a possible indication of the radioprotective properties of A1M in renal tissue. Since our results showed an upregulation of *TNFRSF10B* after irradiation in GOT1 tumors at both time points, regardless of A1M administration, it seems likely that A1M can affect normal and tumor tissues differently.

Regarding the response after exposure to A1M alone, no statistically significant regulated genes were detected after 1 day, but among the genes with high expression, *CASP10*, *GADD45A* and *TP73* were all significantly upregulated, two of which had significantly higher FC values than for both irradiated groups. After 7 days, a similar transcription pattern as for irradiated mice was found, with some important exceptions. For example, statistically significant upregulation was detected for the *CASP3* and CRADD genes, and downregulation of the CASP8, *DFFA*, *TNFRSF10B* and *IGF1R* genes, compared to the irradiated groups. Thus, A1M alone may induce some pro-apoptotic responses early, whereas the later response at day 7 consists of both pro- and anti-apoptotic responses. Further studies are necessary to determine the long-term effects of A1M in tumor tissue.

In this work we focused on effects related to apoptosis, since it has been a widely studied cell death mechanism, and frequently been connected to radiobiological effects and radiation therapy^33^. Furthermore, we have previously confirmed that apoptosis was prominent in similar experiments on GOT1 and ^177^Lu-octreotate during the first days after injection^32^. In general, upregulated pro-apoptotic and down-regulated anti-apoptotic genes could indicate an anti-survival response, whereas the opposite indicates a pro-survival response. However, the apoptosis mechanisms are very complex, and include interactions between proteins that may act either pro- or anti-apoptotic or both (Supplementary Table S1). Apoptosis consists of two main pathways, the extrinsic and the intrinsic pathways. A third pathway is the perforin/granzyme pathway. Each of these pathways activates an initiator caspase unique for the pathway: caspase 8, 9 and 10 for the extrinsic, intrinsic and one of the perforin pathways, respectively. These initiators will then all activate the executor caspase 3, leading to apoptotic body formation^34^. It has been proposed that radiation mainly acts via the intrinsic pathway, based on in vitro studies^35^. In the present study the regulation of these initiator caspases was not statistically significant in the ^177^Lu-octreotate and ^177^Lu-octreotate + A1M groups. However, *CASP10* was highly upregulated in the A1M group (based on data from only one tumor), indicating that A1M may activate the perforin/granzyme B pathway. We also found that genes related to the extrinsic pathway were regulated in the groups treated with ^177^Lu-octreotate, maybe demonstrating differences between in vitro and in vivo studies, including physiologic and metabolic factors from tumor microenvironment and the rest of the organism.

Furthermore, apoptosis may be suppressed during cancer development, and resistance to apoptosis can be acquired by tumor cells, e.g., related to expression of BAX or B-cell lymphoma 2 (BCL2), two major pro and anti-apoptotic proteins from the same family and regulated by the *TP53* tumor suppressor gene. *BAX* prevents *BCL2* from inhibiting apoptosis, and *BCL2* prevents *BAX* from initiating the intrinsic apoptotic pathway^26,34,36^. The ratio between the expression levels of BAX and BCL2 may indicate susceptibility to apoptosis^37^. In this study, none of the treatment groups demonstrated a significant regulation of *BCL2*, which suggests an imbalance in GOT1 between these primary apoptosis regulators in favor of the pro-apoptotic marker *BAX*.

The *TP53* gene is interesting in the context of radiation-induced cell damage. In the presence of DNA damage, the *TP53* gene is activated, which results in transcription of genes that regulate cell cycle arrest, DNA repair, and apoptosis. Many cancers have mutated *TP53* that can contribute to inhibition of apoptosis. However, *TP53* mutations are rare in many NETs^20^, including the GOT1 tumor model. Thus, activation of *TP53* should be expected. In this study, the *TP53* expression was not significantly altered in any groups or time-points. One reason for this could be that the mRNA has already been translated or degraded when the tumors were analyzed. We have previously shown that several genes involved in p53 signaling, related to both growth arrest and apoptosis, were regulated in GOT1 tumor model after exposure to 15 MBq ^177^Lu-octreotate^38^. These genes include *BAX* and *Bcl2 Interacting Protein 3* (*BNIP3)*, which were also upregulated in the present study. *TP73*, with significant structural and functional similarities to *TP53*, can transactivate the TP53 pathway-related target genes and is suggested to be a tumor suppressor gene^39^. Overexpression of *TP73* can inhibit cell growth and induce apoptosis by activation of TP53-responsive genes in neuroblastomas, but its role in other types of NETs, such as GOT1, is still unknown^40^.

In our previous study on effects on tumor volume, injection of 30 MBq ^177^Lu-octreotate with and without A1M reduced GOT1 tumor volume to almost 30% after 7 days in a similar way^9^. Furthermore, no tumor volume reduction was obtained after administration of A1M only. This agrees with the present study, where administration with A1M resulted in a similar increase in tumor volume as controls after 7 days. In groups exposed to ^177^Lu-octreotate, there was a similar tumor growth at 7 days regardless of co-administration with A1M. Altogether, this indicates that A1M does not reduce the early anti-tumor effect of ^177^Lu-octreotate on GOT1 tumors. Gene expression analysis in the present study indicates that cell death via apoptosis may contribute to the volume reduction and that combination with A1M does not mitigate this effect. Accordingly, A1M may be used as a radioprotector of kidneys to optimize treatment of NET by allowing higher activity levels administered to patients or include patients previously excluded due to renal insufficiency. Co-administration with A1M and ^177^Lu-octreotate generates an expression profile strongly similar to treatment without A1M after both 1 and 7 days. Thus, A1M does not act as a radioprotector in GOT1 tumors in the short term. However, the late effects of A1M on tumor and various administration schemes need to be further studied to determine the optimum timing of A1M administration and long-term influence of A1M in tumor as well as in normal tissue.

In the present study we used a pre-designed RT-PCR array for gene expression analysis. Of the 84 studied genes, no data could be obtained for 11 of them, and data are missing for at least one group for nine more genes. Furthermore, the amount of data in the groups was reduced for some transcripts, which influenced the statistical results. The reason for missing data can be related to technical issues during the RT-PCR reading, unspecific primer binding or pipetting errors. Results from genes with incomplete data was thus reported separately.

Another factor that should be considered is the potential heterogeneity in somatostatin receptor (SSTR) expression in the GOT1 tumor model^16,38,41,42^. The SSTR expression may be low in a few mice, resulting in lower binding and internalization of ^177^Lu-octreotate, and hence lower absorbed dose to the tumor. Since radiobiological effects, such as apoptosis and cell survival, are dose-dependent, gene expression might be altered. In the present study SSTR2 expression was high in all tumors. Although this animal model has some limitations when it comes to experimental planning, we preferred to make the present study in the GOT1 model, since it is the only transplantable human NET cell line with retained SSTR expression and neuroendocrine properties that is known to us^13,14,43^. This choice, however, limited the number of animals that could be included in the study, together with problems to receive the radiopharmaceutical for preclinical research at the time of experiment. We observed heterogeneity in expression of some of the highly regulated genes, and therefore showed median instead of mean values, and included these data in the interpretation. Nevertheless, despite the mentioned limitations, the results from these predetermined apoptosis related genes seem clear enough making it possible to answer the aims of the study. However, further investigations are required to confirm and validate these results on the protein level, preferably also in in vivo studies on other tumor types.

In conclusion, radiation-induced regulation of expression of genes related to apoptosis was detected on the transcriptional level in GOT1 tumors 1 and 7 days after ^177^Lu-octreotate treatment. Co-treatment with A1M seems not to negatively affect these effects. Administration of A1M alone did not significantly affect apoptotic gene regulation, although potential late effects should be studied further. The results indicate that A1M may be an option for radioprotection of normal tissues in ^177^Lu-octreotate treatment of patients with NET, since no radioprotection was found in tumor tissue.

## Supplementary Information

Supplementary Information.

## Data Availability

The data that support the findings in this study have been deposited in NCBI’s Gene Expression Omnibus and are accessible through GEO Series accession number GSE-200449 (https://www.ncbi.nlm.nih.gov/geo/query/acc.cgi?acc=GSE200449).
