# Supplementary material for: Co-administration with A1M does not influence apoptotic response of 177Lu-octreotate in GOT1 neuroendocrine tumors
Source: Sci Rep. 2023 Apr 19;13:6417. doi: 10.1038/s41598-023-32091-9 (PMC10115890; doi:10.1038/s41598-023-32091-9)
Supplement: Supplementary file 1 — Supplementary Information. [file 41598_2023_32091_MOESM1_ESM.pdf]

# Supplementary Information

**Supplementary Table 1**. GO annotations related to cell death for genes with statistically significant expression in GOT1 tissue at one or seven days following injection with $^{177}$Lu-octreotate, $^{177}$Lu-octreotate + A1M or A1M.

| Gene abbreviation and description | GO terms related to the biological process of cell death in human tissue |
|---|---|
| *BAX* (BCL2 Associated X) | apoptotic mitochondrial changes |
| | apoptotic process |
| | apoptotic process involved in blood vessel morphogenesis |
| | apoptotic process involved in embryonic digit morphogenesis |
| | apoptotic signaling pathway |
| | B cell apoptotic process |
| | B cell receptor apoptotic signaling pathway |
| | ectopic germ cell programmed cell death |
| | extrinsic apoptotic signaling pathway |
| | extrinsic apoptotic signaling pathway in absence of ligand |
| | extrinsic apoptotic signaling pathway via death domain receptors |
| | intrinsic apoptotic signaling pathway |
| | intrinsic apoptotic signaling pathway by p53 class mediator |
| | intrinsic apoptotic signaling pathway in response to DNA damage |
| | intrinsic apoptotic signaling pathway in response to endoplasmic reticulum stress |
| | mitochondrial fragmentation involved in apoptotic process |
| | negative regulation of apoptotic signaling pathway |
| | negative regulation of neuron apoptotic process |
| | neuron apoptotic process |
| | positive regulation of apoptotic DNA fragmentation |
| | positive regulation of apoptotic process |
| | positive regulation of apoptotic process involved in mammary gland involution |
| | positive regulation of B cell apoptotic process |
| | positive regulation of extrinsic apoptotic signaling pathway in absence of ligand |
| | positive regulation of intrinsic apoptotic signaling pathway |
| | positive regulation of mitochondrial membrane permeability involved in apoptotic process |
| | positive regulation of neuron apoptotic process |
| | positive regulation of release of cytochrome c from mitochondria |
| | protein insertion into mitochondrial membrane involved in apoptotic signaling pathway |
| | regulation of apoptotic process |
| | regulation of mitochondrial membrane permeability involved in apoptotic process |
| | release of cytochrome c from mitochondria |
| | release of matrix enzymes from mitochondria |
| | retinal cell programmed cell death |

| | |
|---|---|
| | thymocyte apoptotic process |
| *BAD* (BCL2 associated agonist of cell death) | apoptotic process |
| | apoptotic signaling pathway |
| | apoptotic signaling pathway |
| | extrinsic apoptotic signaling pathway |
| | extrinsic apoptotic signaling pathway in absence of ligand |
| | intrinsic apoptotic signaling pathway |
| | intrinsic apoptotic signaling pathway in response to DNA damage |
| | positive regulation of apoptotic process |
| | positive regulation of apoptotic process by virus |
| | positive regulation of granulosa cell apoptotic process |
| | positive regulation of intrinsic apoptotic signaling pathway in response to osmotic stress |
| | positive regulation of release of cytochrome c from mitochondria |
| | regulation of apoptotic process |
| | release of cytochrome c from mitochondria |
| | suppression by virus of host apoptotic process |
| *BAK1* (BCL2 Antagonist/Killer 1) | apoptotic process |
| | apoptotic process involved in blood vessel morphogenesis |
| | apoptotic signaling pathway |
| | B cell apoptotic process |
| | extrinsic apoptotic signaling pathway in absence of ligand |
| | extrinsic apoptotic signaling pathway in absence of ligand |
| | extrinsic apoptotic signaling pathway in absence of ligand |
| | fibroblast apoptotic process |
| | intrinsic apoptotic signaling pathway in response to DNA damage |
| | intrinsic apoptotic signaling pathway in response to endoplasmic reticulum stress |
| | intrinsic apoptotic signaling pathway in response to endoplasmic reticulum stress |
| | positive regulation of apoptotic process |
| | positive regulation of release of cytochrome c from mitochondria |
| | regulation of apoptotic process |
| | release of cytochrome c from mitochondria |
| | thymocyte apoptotic process |
| *CASP6* (Caspase 6) | apoptotic process |
| | hepatocyte apoptotic process |
| | intrinsic apoptotic signaling pathway by p53 class mediator |
| | positive regulation of apoptotic process |
| | positive regulation of necroptotic process |
| | positive regulation of neuron apoptotic process |
| | positive regulation of retinal cell programmed cell death |
| | Pyroptosis |
| | regulation of apoptotic process |
| *FAS* (Fas Cell Surface Death Receptor) | activation-induced cell death of T cells |
| | apoptotic process |
| | apoptotic signaling pathway |
| | extrinsic apoptotic signaling pathway |
| | extrinsic apoptotic signaling pathway in absence of ligand |

| | |
|---|---|
| | extrinsic apoptotic signaling pathway via death domain receptors |
| | hepatocyte apoptotic process |
| | inflammatory cell apoptotic process |
| | lymphocyte apoptotic process |
| | motor neuron apoptotic process |
| | necroptotic signaling pathway |
| | negative regulation of apoptotic process |
| | neuron apoptotic process |
| | positive regulation of apoptotic process |
| | positive regulation of apoptotic signaling pathway |
| | positive regulation of neuron apoptotic process |
| | positive regulation of release of cytochrome c from mitochondria |
| | regulation of apoptotic process |
| *TNFRSF10B* (TNF Receptor Superfamily Member 10b) | apoptotic process |
| | extrinsic apoptotic signaling pathway via death domain receptors |
| | intrinsic apoptotic signaling pathway in response to endoplasmic reticulum stress |
| | positive regulation of apoptotic process |
| | regulation of apoptotic process |
| | TRAIL-activated apoptotic signaling pathway |
| *CASP3* (Caspase 3) | apoptotic process |
| | apoptotic signaling pathway |
| | execution phase of apoptosis |
| | extrinsic apoptotic signaling pathway |
| | extrinsic apoptotic signaling pathway via death domain receptors |
| | glial cell apoptotic process |
| | hepatocyte apoptotic process |
| | intrinsic apoptotic signaling pathway in response to endoplasmic reticulum stress |
| | intrinsic apoptotic signaling pathway in response to osmotic stress |
| | negative regulation of apoptotic process |
| | neuron apoptotic process |
| | negative regulation of necroptotic process |
| | positive regulation of apoptotic DNA fragmentation |
| | positive regulation of apoptotic process |
| | positive regulation of extrinsic apoptotic signaling pathway |
| | positive regulation of neuron apoptotic process |
| | pyroptosis |
| | regulation of apoptotic process |
| | regulation of apoptotic signaling pathway |
| | regulation of thymocyte apoptotic process |
| | TRAIL-activated apoptotic signaling pathway |
| *DAPK1* (Death Associated Protein Kinase 1) | apoptotic process |
| | apoptotic signaling pathway |
| | extrinsic apoptotic signaling pathway via death domain receptors |
| | negative regulation of apoptotic process |

| | |
|---|---|
| | negative regulation of extrinsic apoptotic signaling pathway via death domain receptors |
| | positive regulation of apoptotic process |
| | positive regulation of autophagic cell death |
| *CRADD* (CASP2 And RIPK1 Domain Containing Adaptor With Death Domain) | apoptotic process |
| | apoptotic signaling pathway |
| | negative regulation of neuron apoptotic process |
| | positive regulation of apoptotic process |
| | positive regulation of apoptotic signaling pathway |
| | regulation of apoptotic process |
| *DFFA* (DNA Fragmentation Factor Subunit Alpha) | apoptotic DNA fragmentation |
| | apoptotic process |
| | negative regulation of apoptotic DNA fragmentation |
| | negative regulation of execution phase of apoptosis |
| | positive regulation of apoptotic process |
| | regulation of apoptotic process |
| | thymocyte apoptotic process |
| *NOD1* (Nucleotide Binding Oligomerization Domain Containing 1) | apoptotic process |
| | regulation of apoptotic process |
| | positive regulation of cell death |
| *BCL2L2* (BCL2 Like 2) | suppression by virus of host apoptotic process |
| | apoptotic process |
| | extrinsic apoptotic signaling pathway in absence of ligand |
| | intrinsic apoptotic signaling pathway in response to DNA damage |
| | negative regulation of apoptotic process |
| | negative regulation of intrinsic apoptotic signaling pathway |
| | negative regulation of release of cytochrome c from mitochondria |
| | regulation of apoptotic process |
| *BIRC2* (Baculoviral IAP Repeat Containing 2) | necroptotic process |
| | negative regulation of apoptotic process |
| | negative regulation of cysteine-type endopeptidase activity involved in apoptotic process |
| | negative regulation of necroptotic process |
| | negative regulation of ripoptosome assembly involved in necroptotic process |
| | regulation of apoptotic process |
| | regulation of necroptotic process |
| *BIRC5* (Baculoviral IAP Repeat Containing 5) | apoptotic process |
| | negative regulation of apoptotic process |
| | negative regulation of cysteine-type endopeptidase activity involved in apoptotic process |
| *BIRC3* (Baculoviral IAP Repeat Containing 3) | apoptotic process |
| | negative regulation of apoptotic process |
| | negative regulation of cysteine-type endopeptidase activity involved in apoptotic process |
| | negative regulation of necroptotic process |
| | regulation of apoptotic process |
| | regulation of necroptotic process |
| *IGF1R* (Insulin Like Growth Factor 1) | extrinsic apoptotic signaling pathway in absence of ligand |
| | negative regulation of apoptotic process |
| | negative regulation of cholangiocyte apoptotic process |

| | |
|---|---|
| | negative regulation of cysteine-type endopeptidase activity involved in apoptotic process |
| | negative regulation of extrinsic apoptotic signaling pathway |
| | negative regulation of muscle cell apoptotic process |
| | negative regulation of oligodendrocyte apoptotic process |
| | negative regulation of release of cytochrome c from mitochondria |
| | negative regulation of smooth muscle cell apoptotic process |
| | regulation of vascular associated smooth muscle cell apoptotic process |
| | negative regulation of hepatocyte apoptotic process |
| | negative regulation of cholangiocyte apoptotic process |
| | negative regulation of hepatocyte apoptotic process |
| *TNFRSF25* (TNF Receptor Superfamily Member 25) | apoptotic signaling pathway |
| | regulation of apoptotic process |
| | apoptotic process |

**Supplementary Table 2.** Results from reactome pathway analysis. Included in the analyses at one and seven days following injection with 177Lu-octreotate were the *BIRC3*, *BAK*, *BAK1* and *BCL2L2* genes (1 day), and the *FAS*, *BIRC2*, and *TNFRSF25* genes (7 days). Similarly, the *FAS*, *TNFRSF10B*, *CASP6* and *BAD* genes were included for the 177Lu-octreotate + A1M group after one day, and the *FADD*, *FAS* and *NOD1* genes after seven days. Lastly, the genes included in the pathway analysis for animals injected with A1M and sacrificed after one day were the *DFFA*, *DAPK1*, *CRADD*, *CASP8*, *TNFRSF10B* and *IGF1R* genes. $p<0.05$

**1 day after injection of $^{177}$Lu-cotreotate**
**Significantly expressed genes: *BAX*, *BAK1*, *BCL2L2*, *BIRC3***

| Biological process | Pathway name | p-value |
|---|---|---|
| Programmed cell death | Regulated necrosis | 3,97E-05 |
| Immune system | TNF receptor superfamily (TNFSF) members mediating non-canonical NF-kB pathway | 4,43E-05 |
| Immune system | TICAM1, RIP1-mediated IKK complex recruitment | 1,13E-04 |
| Immune system | IKK complex recruitment mediated by RIP1 | 1,43E-04 |
| Signal transduction | TNFR1-induced proapoptotic signaling | 3,34E-04 |
| Programmed cell death | Release of apoptotic factors from the mitochondria | 3,34E-04 |
| Programmed cell death | Pyroptosis | 5,91E-04 |
| Gene expression (transcription) | TP53 regulates transcription of genes Involved in cytochrome C release | 1,92E-03 |
| Programmed cell death | Apoptotic factor-mediated response | 2,17E-03 |
| Programmed cell death | Programmed cell death | 2,93E-03 |
| Immune system | NOD1/2 signaling pathway | 2,98E-03 |
| Immune system | TNFR2 non-canonical NF-kB pathway | 4,32E-03 |
| Programmed cell death | Activation of NOXA and translocation to mitochondria | 4,39E-03 |
| Programmed cell death | Activation of PUMA and translocation to mitochondria | 5,99E-03 |

| | | |
|---|---|---|
| Programmed cell death | Intrinsic pathway for apoptosis | 7,45E-03 |
| Signal transduction | TNFR1-induced NFkappaB signaling pathway | 7,89E-03 |
| Programmed cell death | Activation, myristolyation of BID and translocation to mitochondria | 8,77E-03 |
| Immune system | The AIM2 inflammasome | 9,17E-03 |
| Metabolism of proteins | Ub-specific processing proteases | 9,84E-03 |
| Programmed cell death | Apoptosis | 1,15E-02 |
| Immune system | CLEC7A/inflammasome pathway | 1,19E-02 |
| Programmed cell death | Activation, translocation and oligomerization of BAX | 1,27E-02 |
| Programmed cell death | Regulation of necroptotic cell death | 1,40E-02 |
| Immune system | Nucleotide-binding domain, leucine rich repeat containing receptor (NLR) signaling pathways | 1,43E-02 |
| Programmed cell death | RIPK1-mediated regulated necrosis | 1,44E-02 |
| Programmed cell death | Activation and oligomerization of BAK protein | 1,47E-02 |
| Programmed cell death | Activation of BAD and translocation to mitochondria | 1,51E-02 |
| Programmed cell death | Activation of BIM and translocation to mitochondria | 1,63E-02 |
| Disease | SARS-CoV-1-mediated effects on programmed cell death | 1,63E-02 |
| Immune system | RIP-mediated NFkB activation via ZBP1 | 1,67E-02 |
| Programmed cell death | Activation of BMF and translocation to mitochondria | 1,79E-02 |
| Signal transduction | NTRK3 as a dependence receptor | 1,94E-02 |
| Gene expression (transcription) | RUNX3 regulates BCL2L11 (BIM) transcription | 1,98E-02 |
| Immune system | Toll Like receptor 3 (TLR3) cascade | 1,99E-02 |
| Gene expression (transcription) | TP53 regulates transcription of cell death genes | 2,20E-02 |
| Immune system | TLR3-mediated TICAM1-dependent programmed cell death | 2,26E-02 |
| Immune system | TRIF(TICAM1)-mediated TLR4 signaling | 2,28E-02 |
| Immune system | MyD88-independent TLR4 cascade | 2,28E-02 |
| Transport of small molecules | VLDL assembly | 2,30E-02 |
| Immune system | TRIF-mediated programmed cell death | 2,42E-02 |
| Disease | Microbial modulation of RIPK1-mediated regulated necrosis | 2,45E-02 |
| Disease | Defective RIPK1-mediated regulated necrosis | 2,45E-02 |
| Programmed cell death | BH3-only proteins associate with and inactivate anti-apoptotic BCL-2 members | 2,57E-02 |
| Immune system | NF-kB activation through FADD/RIP-1 pathway mediated by caspase-8 and -10 | 3,00E-02 |
| Gene expression (transcription) | Transcriptional regulation by RUNX2 | 3,13E-02 |
| Immune system | Toll-like receptor 4 (TLR4) cascade | 3,29E-02 |
| Disease | Nuclear events stimulated by ALK signaling in cancer | 3,47E-02 |
| Signal transduction | RAS processing | 3,55E-02 |
| Immune system | ZBP1(DAI) mediated induction of type I IFNs | 3,74E-02 |
| Programmed cell death | Activation of BH3-only proteins | 3,93E-02 |
| Programmed cell death | Regulation of the apoptosome activity | 4,16E-02 |
| Metabolism of proteins | Pyruvate metabolism | 4,20E-02 |
| Immune system | Toll-like receptor cascades | 4,27E-02 |
| Transport of small molecules | Plasma lipoprotein assembly | 4,51E-02 |
| Disease | STAT5 activation downstream of FLT3 ITD mutants | 4,74E-02 |

## 7 days after injection of $^{177}$Lu-cotreotate
## Significantly expressed genes: *FAS, BIRC2, TNFRSF25*

| Biological process | Pathway name | p-value |
|---|---|---|
| Immune system | TNF receptor superfamily (TNFSF) members mediating non-canonical NF-kB pathway | 5,53E-05 |
| Gene expression (transcription) | TP53 regulates transcription of death receptors and ligands | 1,41E-04 |
| Immune system | TICAM1, RIP1-mediated IKK complex recruitment | 1,41E-04 |
| Immune system | TNFR2 non-canonical NF-kB pathway | 1,61E-04 |
| Immune system | IKK complex recruitment mediated by RIP1 | 1,78E-04 |
| Signal transduction | TNFR1-induced proapoptotic signaling | 4,17E-04 |
| Signal transduction | NR1H2 & NR1H3 regulate gene expression linked to lipogenesis | 6,90E-04 |
| Programmed cell death | Regulation of necroptotic cell death | 9,57E-04 |
| Programmed cell death | RIPK1-mediated regulated necrosis | 9,94E-04 |
| Programmed cell death | Regulated necrosis | 1,51E-03 |
| Metabolism | Activation of gene expression by SREBF (SREBP) | 3,33E-03 |
| Immune system | NOD1/2 signaling pathway | 3,70E-03 |
| Metabolism | Regulation of cholesterol biosynthesis by SREBP (SREBF) | 5,20E-03 |
| Programmed cell death | CASP8 activity is inhibited | 5,77E-03 |
| Signal transduction | TNFR1-induced NFkappaB signaling pathway | 9,76E-03 |
| Metabolism | ChREBP activates metabolic gene expression | 1,02E-02 |
| Metabolism of proteins | Ub-specific processing proteases | 1,22E-02 |
| Programmed cell death | Dimerization of procaspase-8 | 1,41E-02 |
| Programmed cell death | Regulation by c-FLIP | 1,41E-02 |
| Signal transduction | NR1H2 and NR1H3-mediated signaling | 1,67E-02 |
| Immune system | Nucleotide-binding domain, leucine rich repeat containing receptor (NLR) signaling pathways | 1,77E-02 |
| Programmed cell death | Caspase activation via death receptors in the presence of ligand | 1,81E-02 |
| Immune system | RIP-mediated NFkB activation via ZBP1 | 1,85E-02 |
| Disease | TRAF3 deficiency - HSE | 1,94E-02 |
| Metabolism | Vitamin B5 (pantothenate) metabolism | 2,29E-02 |
| Immune system | Toll-like receptor 3 (TLR3) cascade | 2,45E-02 |
| Immune system | TLR3-mediated TICAM1-dependent programmed cell death | 2,51E-02 |
| Signal transduction | Death receptor signaling | 2,59E-02 |
| Immune system | TRIF-mediated programmed cell death | 2,68E-02 |
| Gene expression (transcription) | TP53 regulates transcription of cell death genes | 2,70E-02 |
| Disease | Microbial modulation of RIPK1-mediated regulated necrosis | 2,72E-02 |
| Disease | Defective RIPK1-mediated regulated necrosis | 2,72E-02 |
| Immune system | TRIF(TICAM1)-mediated TLR4 signaling | 2,80E-02 |
| Immune system | MyD88-independent TLR4 cascade | 2,80E-02 |
| Immune system | TICAM1-dependent activation of IRF3/IRF7 | 2,85E-02 |
| Cell cycle | Polymerase switching on the C-strand of the telomere | 2,94E-02 |
| Programmed cell death | Release of apoptotic factors from the mitochondria | 3,03E-02 |
| Immune system | NF-kB activation through FADD/RIP-1 pathway mediated by caspase-8 and -10 | 3,33E-02 |
| Programmed cell death | Programmed cell death | 3,39E-02 |

| | | |
|---|---|---|
| Signal transduction | TRAIL signaling | 3,54E-02 |
| Disease | Defective inhibition of DNA recombination at telomere due to ATRX mutations | 3,59E-02 |
| Disease | Alternative lengthening of telomeres (ALT) | 3,72E-02 |
| Disease | Defective inhibition of DNA recombination at telomere | 3,72E-02 |
| Disease | Diseases of telomere maintenance | 3,72E-02 |
| Immune system | TRAF3-dependent IRF activation pathway | 3,72E-02 |
| Gene expression (transcription) | RUNX1 regulates transcription of genes involved in BCR signaling | 3,76E-02 |
| Immune system | TNFs bind their physiological receptors | 3,76E-02 |
| Signal transduction | FasL/ CD95L signaling | 3,97E-02 |
| Immune system | Toll-like receptor 4 (TLR4) Cascade | 4,03E-02 |
| Immune system | TRAF6 mediated IRF7 activation | 4,06E-02 |
| Immune system | ZBP1(DAI) mediated induction of type I IFNs | 4,15E-02 |
| Disease | IKBKB deficiency causes SCID | 4,19E-02 |
| Hemostasis | Basigin interactions | 4,27E-02 |
| Signal transduction | Regulation of PTEN localization | 4,53E-02 |
| Cell-cell communication | Localization of the PINCH-ILK-PARVIN complex to focal adhesions | 4,62E-02 |
| Programmed cell death | Regulation of the apoptosome activity | 4,62E-02 |
| Immune system | Activation of IRF3/IRF7 mediated by TBK1/IKK epsilon | 4,83E-02 |
| Disease | SARS-CoV-2 targets host intracellular signaling and regulatory pathways | 4,87E-02 |

## 1 day after injection of $^{177}$Lu-cotreotate + A1M
## Significantly expressed genes: *BAD, CASP6, FAS, TNFRSF10B*

| Biological process | Pathway name | p-value |
|---|---|---|
| Gene expression (transcription) | TP53 regulates transcription of death receptors and ligands | 2,27E-11 |
| Gene expression (transcription) | TP53 regulates transcription of cell death genes | 1,29E-08 |
| Programmed cell death | CASP8 activity is inhibited | 2,60E-05 |
| Gene expression (transcription) | transcriptional regulation by TP53 | 6,17E-05 |
| Programmed cell death | Dimerization of procaspase-8 | 1,56E-04 |
| Programmed cell death | Regulation by c-FLIP | 1,56E-04 |
| Programmed cell death | Caspase activation via death receptors in the presence of ligand | 2,56E-04 |
| Signal transduction | TRAIL signaling | 9,86E-04 |
| Signal transduction | NR1H2 & NR1H3 regulate gene expression linked to lipogenesis | 1,19E-03 |
| Programmed cell death | Apoptosis | 4,72E-03 |
| Metabolism | Activation of gene expression by SREBF (SREBP) | 5,67E-03 |
| Programmed cell death | Caspase activation via extrinsic apoptotic signaling pathway | 5,78E-03 |
| Metabolism | Regulation of cholesterol biosynthesis by SREBP (SREBF) | 8,82E-03 |
| Signal transduction | Death receptor signaling | 9,14E-03 |
| Gene expression (transcription) | TP53 regulates transcription of caspase activators and caspases | 1,17E-02 |
| Programmed cell death | Programmed cell death | 1,30E-02 |
| Metabolism | ChREBP activates metabolic gene expression | 1,32E-02 |
| Programmed cell death | Breakdown of the nuclear lamina | 1,49E-02 |
| Metabolism | Methionine salvage pathway | 1,95E-02 |

| | | |
|---|---|---|
| Programmed cell death | Activation of BAD and translocation to mitochondria | 2,17E-02 |
| Disease | SARS-CoV-1-mediated effects on programmed cell death | 2,34E-02 |
| Programmed cell death | Activation of BIM and translocation to mitochondria | 2,34E-02 |
| Transport of small molecules | Cellular hexose transport | 2,34E-02 |
| Signal transduction | NR1H2 and NR1H3-mediated signaling | 2,78E-02 |
| Gene expression (transcription) | RUNX3 regulates BCL2L11 (BIM) transcription | 2,85E-02 |
| Programmed cell death | Regulation of necroptotic cell death | 2,88E-02 |
| Programmed cell death | RIPK1-mediated regulated necrosis | 2,95E-02 |
| Metabolism | Vitamin B5 (pantothenate) metabolism | 2,96E-02 |
| Cell cycle | Telomere C-strand synthesis initiation | 3,02E-02 |
| Gene expression (transcription) | Generic transcription pathway | 3,19E-02 |
| Immune system | TLR3-mediated TICAM1-dependent programmed cell death | 3,25E-02 |
| Vesicle-mediated transport | Endosomal sorting complex required for transport (ESCRT) | 3,36E-02 |
| Developmental biology | Role of second messengers in netrin-1 signaling | 3,41E-02 |
| Immune system | TRIF-mediated programmed cell death | 3,47E-02 |
| Disease | Microbial modulation of RIPK1-mediated regulated necrosis | 3,53E-02 |
| Disease | Defective RIPK1-mediated regulated necrosis | 3,53E-02 |
| Transport of small molecules | Chylomicron assembly | 3,53E-02 |
| Programmed cell death | BH3-only proteins associate with and inactivate anti-apoptotic BCL-2 members | 3,69E-02 |
| Developmental biology | Transcriptional regulation of testis differentiation | 3,75E-02 |
| Gene expression (transcription) | RNA polymerase II transcription | 3,81E-02 |
| Programmed cell death | Regulated Necrosis | 3,85E-02 |
| Signal transduction | TNFR1-induced proapoptotic signaling | 3,92E-02 |
| Immune system | NF-kB activation through FADD/RIP-1 pathway mediated by caspase-8 and -10 | 4,31E-02 |
| Metabolism | Synthesis of prostaglandins (PG) and thromboxanes (TX) | 4,31E-02 |
| Disease | Defective inhibition of DNA recombination at telomere due to ATRX mutations | 4,64E-02 |
| Disease | Defective Inhibition of DNA recombination at telomere | 4,80E-02 |
| Disease | Diseases of telomere maintenance | 4,80E-02 |
| Disease | Alternative lengthening of telomeres (ALT) | 4,80E-02 |
| Gene expression (transcription) | Gene expression (transcription) | 4,83E-02 |
| Cellular response to stimuli | NFE2L2 regulating anti-oxidant/detoxification enzymes | 4,86E-02 |
| Metabolism | Synthesis of PG | 4,97E-02 |

## 7 days after injection of $^{177}$Lu-cotreotate + A1M
## Significantly expressed genes: *FADD, FAS, NOD1, BIRC5*

| Biological process | Pathway name | p-value |
|---|---|---|
| Programmed cell death | CASP8 activity is inhibited | 3,99E-05 |
| Programmed cell death | Dimerization of procaspase-8 | 2,40E-04 |
| Programmed cell death | Regulation by c-FLIP | 2,40E-04 |
| Gene expression (transcription) | TP53 regulates transcription of death receptors and ligands | 3,73E-04 |

| | | |
|---|---|---|
| Programmed cell death | Caspase activation via death receptors in the presence of ligand | 3,92E-04 |
| Gene expression (transcription) | TP53 regulates transcription of cell death genes | 6,74E-04 |
| Signal transduction | NR1H2 & NR1H3 regulate gene expression linked to lipogenesis | 1,81E-03 |
| Signal transduction | FasL/ CD95L signaling | 1,89E-03 |
| Gene expression (transcription) | TP53 regulates transcription of several additional cell death genes whose specific roles in p53-dependent apoptosis remain uncertain | 5,41E-03 |
| Programmed cell death | Activation of NOXA and translocation to mitochondria | 7,80E-03 |
| Metabolism | Activation of gene expression by SREBF (SREBP) | 8,57E-03 |
| Programmed cell death | Caspase activation via extrinsic apoptotic signaling pathway | 8,74E-03 |
| Programmed cell death | Activation of PUMA and translocation to mitochondria | 1,06E-02 |
| Metabolism | Regulation of cholesterol biosynthesis by SREBP (SREBF) | 1,33E-02 |
| Programmed cell death | Activation, myristolyation of BID and translocation to mitochondria | 1,55E-02 |
| Metabolism | ChREBP activates metabolic gene expression | 1,62E-02 |
| Immune system | JNK (c-Jun kinases) phosphorylation and activation mediated by activated human TAK1 | 1,83E-02 |
| Immune system | activated TAK1 mediates p38 MAPK activation | 1,90E-02 |
| Cell cycle | Interaction between PHLDA1 and AURKA | 2,04E-02 |
| Programmed cell death | Activation, translocation and oligomerization of BAX | 2,25E-02 |
| Programmed cell death | Activation and oligomerization of BAK protein | 2,60E-02 |
| Immune system | Interleukin-4 and Interleukin-13 signaling | 2,65E-02 |
| Programmed cell death | Activation of BAD and translocation to mitochondria | 2,67E-02 |
| Immune system | TICAM1, RIP1-mediated IKK complex recruitment | 2,81E-02 |
| Programmed cell death | Activation of BIM and translocation to mitochondria | 2,88E-02 |
| Immune system | RIP-mediated NFkB activation via ZBP1 | 2,95E-02 |
| DNA Repair | Displacement of DNA glycosylase by APEX1 | 2,95E-02 |
| Programmed cell death | Activation of BMF and translocation to mitochondria | 3,15E-02 |
| Immune system | IKK complex recruitment mediated by RIP1 | 3,15E-02 |
| Disease | MyD88 deficiency (TLR2/4) | 3,22E-02 |
| Immune system | DEx/H-box helicases activate type I IFN and inflammatory cytokines production | 3,36E-02 |
| Signal transduction | NTRK3 as a dependence receptor | 3,43E-02 |
| Gene expression (transcription) | RUNX3 regulates BCL2L11 (BIM) transcription | 3,50E-02 |
| Metabolism | Vitamin B5 (pantothenate) metabolism | 3,64E-02 |
| Cell Cycle | APC/C:Cdc20 mediated degradation of cyclin B | 3,91E-02 |
| Immune system | TLR3-mediated TICAM1-dependent programmed cell death | 3,98E-02 |
| Transport of small molecules | VLDL assembly | 4,05E-02 |
| Signal transduction | NR1H2 and NR1H3-mediated signaling | 4,10E-02 |
| Programmed cell death | Regulation of necroptotic cell death | 4,25E-02 |
| Immune system | TRIF-mediated programmed cell death | 4,25E-02 |
| Disease | Defective RIPK1-mediated regulated necrosis | 4,32E-02 |
| Disease | Microbial modulation of RIPK1-mediated regulated necrosis | 4,32E-02 |
| Programmed cell death | RIPK1-mediated regulated necrosis | 4,35E-02 |
| Programmed cell death | BH3-only proteins associate with and inactivate anti-apoptotic BCL-2 members | 4,53E-02 |
| Disease | NEP/NS2 Interacts with the cellular export machinery | 4,59E-02 |
| Signal transduction | TNFR1-induced proapoptotic signaling | 4,80E-02 |

## 7 days after injection of A1M
## Significantly expressed genes: CASP3, *CASP8, CRADD, DFFA, DAPK1, TNFRSF10B, IGF1R*

| Biological process | Pathway name | p-value |
|---|---|---|
| Signal transduction | TRAIL signaling | 1,83E-14 |
| Signal transduction | Death receptor signaling | 9,10E-10 |
| Gene expression (transcription) | TP53 regulates transcription of cell death genes | 2,15E-09 |
| Programmed cell death | Caspase activation via extrinsic apoptotic signaling pathway | 6,34E-09 |
| Programmed cell death | Apoptosis | 1,38E-07 |
| Gene expression (transcription) | TP53 regulates transcription of death receptors and ligands | 2,70E-07 |
| Programmed cell death | Programmed cell death | 3,89E-07 |
| Signal transduction | FasL/ CD95L signaling | 5,91E-06 |
| Programmed cell death | Apoptotic execution phase | 7,19E-06 |
| Gene expression (transcription) | Transcriptional regulation by TP53 | 1,27E-05 |
| Programmed cell death | Regulated necrosis | 1,99E-05 |
| Programmed cell death | Regulation by c-FLIP | 2,85E-05 |
| Programmed cell death | Dimerization of procaspase-8 | 2,85E-05 |
| Programmed cell death | CASP8 activity is inhibited | 3,39E-05 |
| Programmed cell death | Caspase-mediated cleavage of cytoskeletal proteins | 3,39E-05 |
| Programmed cell death | Caspase activation via dependence receptors in the absence of ligand | 3,39E-05 |
| Programmed cell death | Apoptosis induced DNA fragmentation | 3,98E-05 |
| Immune system | NF-kB activation through FADD/RIP-1 pathway mediated by caspase-8 and -10 | 4,62E-05 |
| Programmed cell death | Caspase activation via death receptors in the presence of ligand | 9,40E-05 |
| Gene expression (transcription) | TP53 regulates transcription of caspase activators and caspases | 9,40E-05 |
| Programmed cell death | Apoptotic cleavage of cellular proteins | 3,37E-04 |
| Programmed cell death | Regulation of necroptotic cell death | 3,55E-04 |
| Programmed cell death | RIPK1-mediated regulated necrosis | 4,71E-04 |
| Programmed cell death | Intrinsic pathway for apoptosis | 9,46E-04 |
| Signal transduction | Signal transduction | 1,89E-03 |
| Immune system | DDX58/IFIH1-mediated induction of interferon-alpha/beta | 2,46E-03 |
| Programmed cell death | Activation, myristolyation of BID and translocation to mitochondria | 2,88E-03 |
| Programmed cell death | Stimulation of the cell death response by PAK-2p34 | 2,88E-03 |
| Gene expression (transcription) | Generic transcription pathway | 3,30E-03 |
| Signal transduction | NADE modulates death signaling | 4,32E-03 |
| Immune system | TLR3-mediated TICAM1-dependent programmed cell death | 4,32E-03 |
| Gene expression (transcription) | RNA polymerase II transcription | 4,82E-03 |
| Programmed cell death | SMAC(DIABLO)-mediated dissociation of IAP:caspase complexes | 5,04E-03 |
| Programmed cell death | SMAC (DIABLO) binds to IAPs | 5,04E-03 |
| Programmed cell death | Activation of caspases through apoptosome-mediated cleavage | 5,76E-03 |
| Programmed cell death | SMAC, XIAP-regulated apoptotic response | 5,76E-03 |

| | | |
|---|---|---|
| Immune system | CLEC7A/inflammasome pathway | 5,76E-03 |
| Gene expression (transcription) | Gene expression (transcription) | 7,13E-03 |
| Immune system | TRIF-mediated programmed cell death | 7,19E-03 |
| Disease | Microbial modulation of RIPK1-mediated regulated necrosis | 7,19E-03 |
| Programmed cell death | Apoptotic cleavage of cell adhesion proteins | 7,91E-03 |
| Disease | Defective RIPK1-mediated regulated necrosis | 9,34E-03 |
| Programmed cell death | Cytochrome c-mediated apoptotic response | 1,08E-02 |
| Signal transduction | SHC-related events triggered by IGF1R | 1,15E-02 |
| Hemostasis | cell surface interactions at the vascular wall | 1,41E-02 |
| Immune system | Regulation of NF-kappa B signaling | 1,43E-02 |
| Signal transduction | Signaling by hippo | 1,58E-02 |
| Programmed cell death | Apoptotic factor-mediated response | 1,65E-02 |
| Immune system | Other interleukin signaling | 1,72E-02 |
| Signal transduction | TNFR1-induced proapoptotic signaling | 1,79E-02 |
| Programmed cell death | Pyroptosis | 2,43E-02 |
| Immune system | NOD1/2 signaling pathway | 2,92E-02 |
| Signal transduction | Regulation of TNFR1 signaling | 3,83E-02 |
| Immune system | TAK1-dependent IKK and NF-kappa-B activation | 3,90E-02 |
| Signal transduction | TNF signaling | 4,52E-02 |
| | IRS-related events triggered by IGF1R | 4,87E-02 |

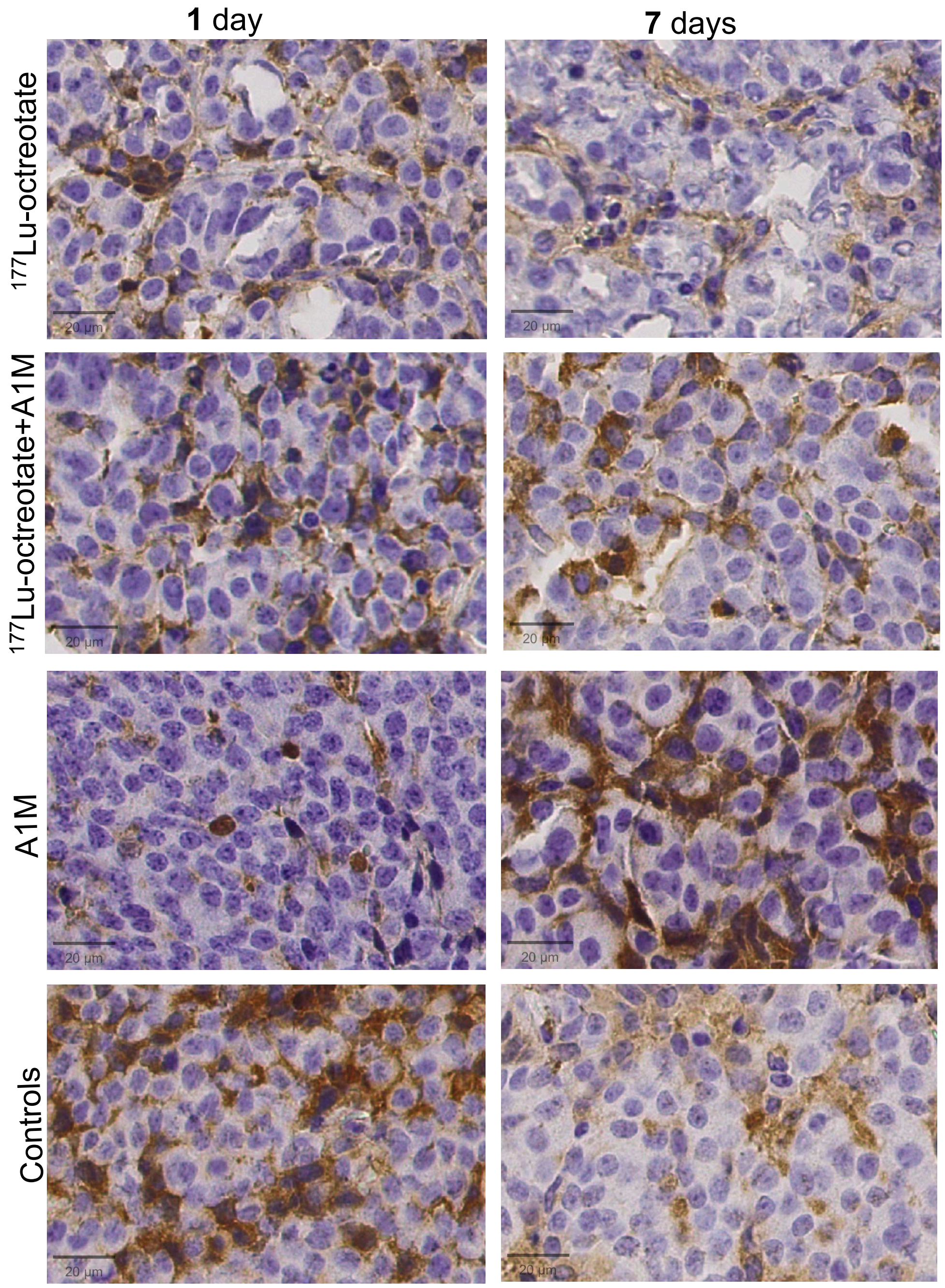


**Supplementary Figure 1.** Typical appearance of GOT1 tumor sections stained against annexin V from mice injected with $^{177}$Lu-octreotate, $^{177}$Lu-octreotate + A1M, A1M and saline (controls), and sacrificed after one and seven days. Black bar=20 µm. Illustrations produced using GraphPad Prism version 9.3.1 for macOS (https://www.graphpad.com/scientific-software/prism/).

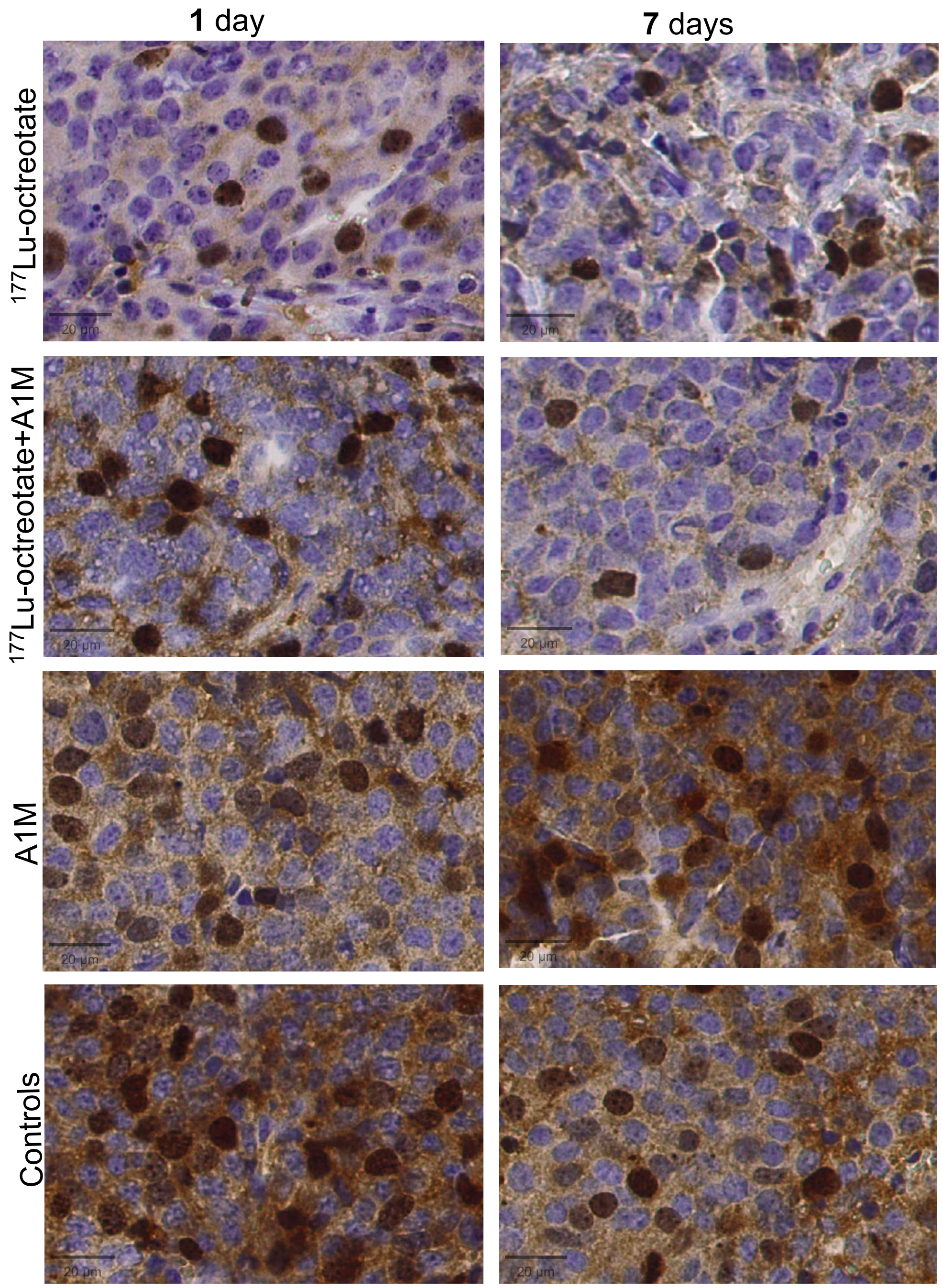


**Supplementary Figure 2.** Typical appearance of GOT1 tumor sections stained against survivin from mice injected with $^{177}$Lu-octreotate, $^{177}$Lu-octreotate + A1M, A1M and saline (controls), and sacrificed after one and seven days. Black bar=20 µm. Illustrations produced using GraphPad Prism version 9.3.1 for macOS (https://www.graphpad.com/scientific-software/prism/).

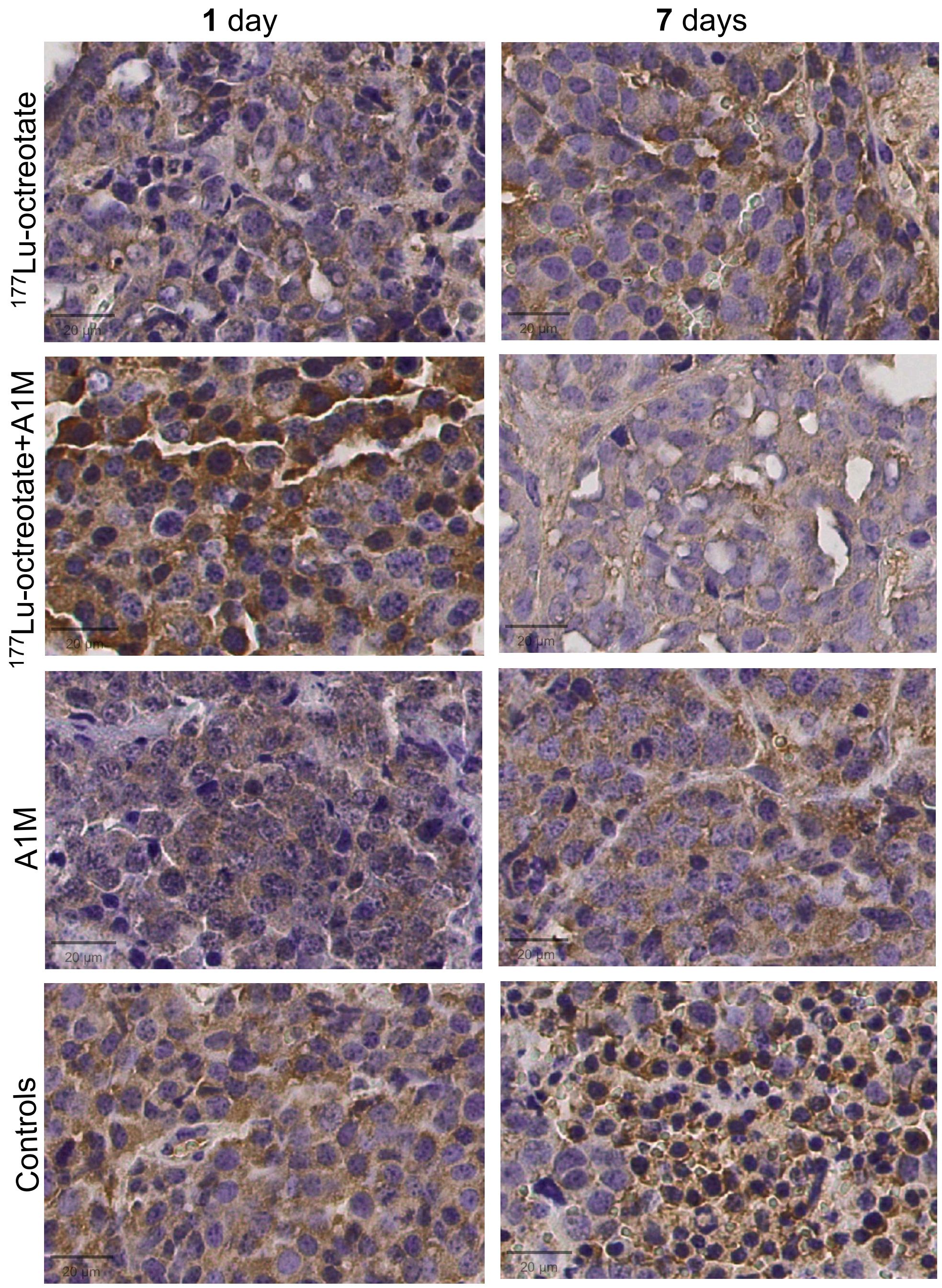


**Supplementary Figure 3.** Typical appearance of GOT1 tumor sections stained against TNFRSF10B from mice injected with $^{177}$Lu-octreotate, $^{177}$Lu-octreotate + A1M, A1M and saline (controls), and sacrificed after one and seven days. Black bar=20 µm. Illustrations produced using GraphPad Prism version 9.3.1 for macOS (https://www.graphpad.com/scientific-software/prism/).

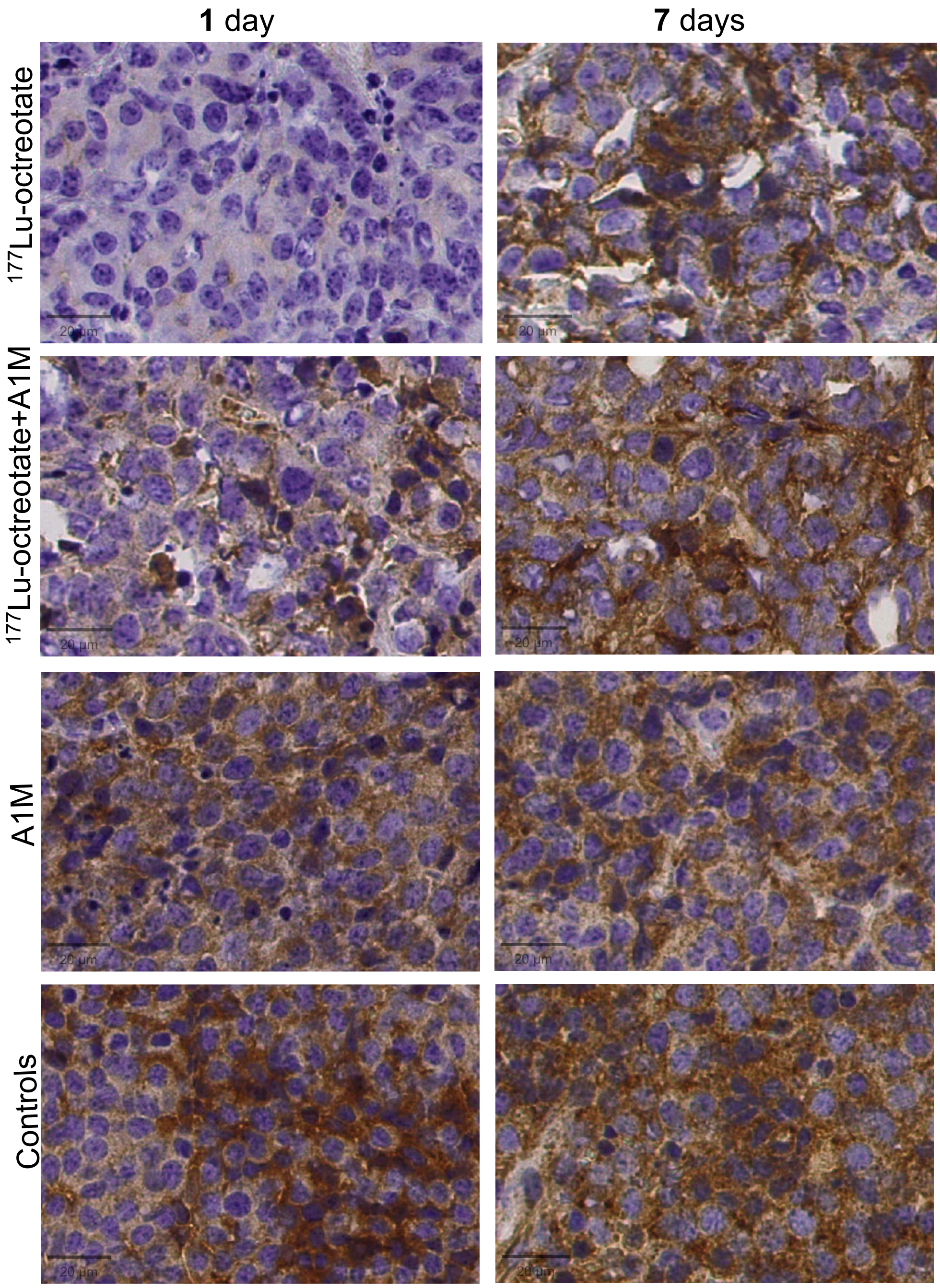


**Supplementary Figure 4.** Typical appearance of GOT1 tumor sections stained against FAS from mice injected with $^{177}$Lu-octreotate, $^{177}$Lu-octreotate + A1M, A1M and saline (controls), and sacrificed after one and seven days. Black bar=20 µm. No positive staining could be observed in tumors from mice injected with $^{177}$Lu-octreotate and sacrificed at one day. Illustrations produced using GraphPad Prism version 9.3.1 for macOS (https://www.graphpad.com/scientific-software/prism/).

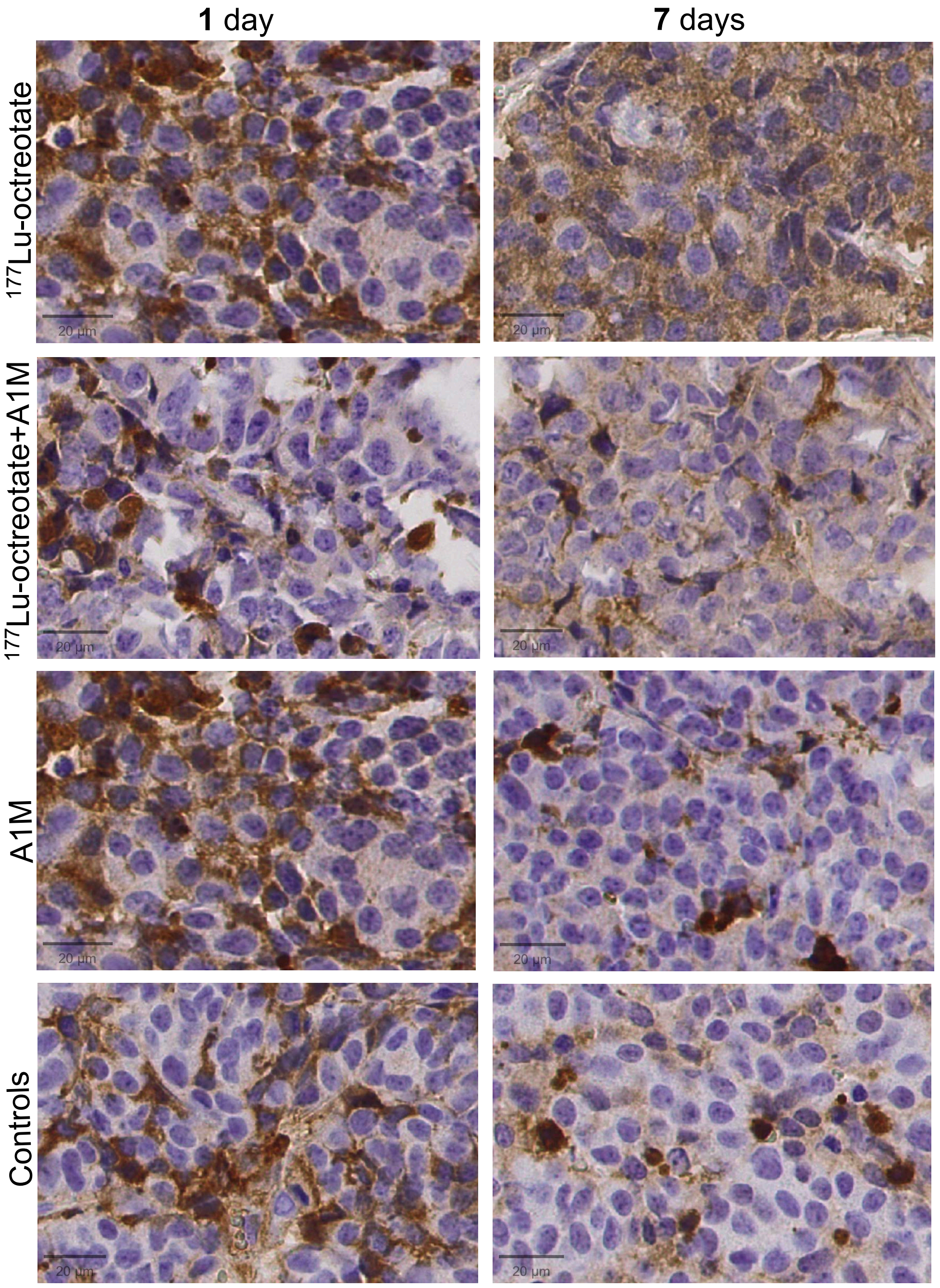


**Supplementary Figure 5.** Typical appearance of GOT1 tumor sections stained against cleaved CASP-3 from mice injected with $^{177}$Lu-octreotate, $^{177}$Lu-octreotate + A1M and saline (controls) and sacrificed after one and seven days. Black bar=20 µm. Illustrations produced using GraphPad Prism version 9.3.1 for macOS (https://www.graphpad.com/scientific-software/prism/).

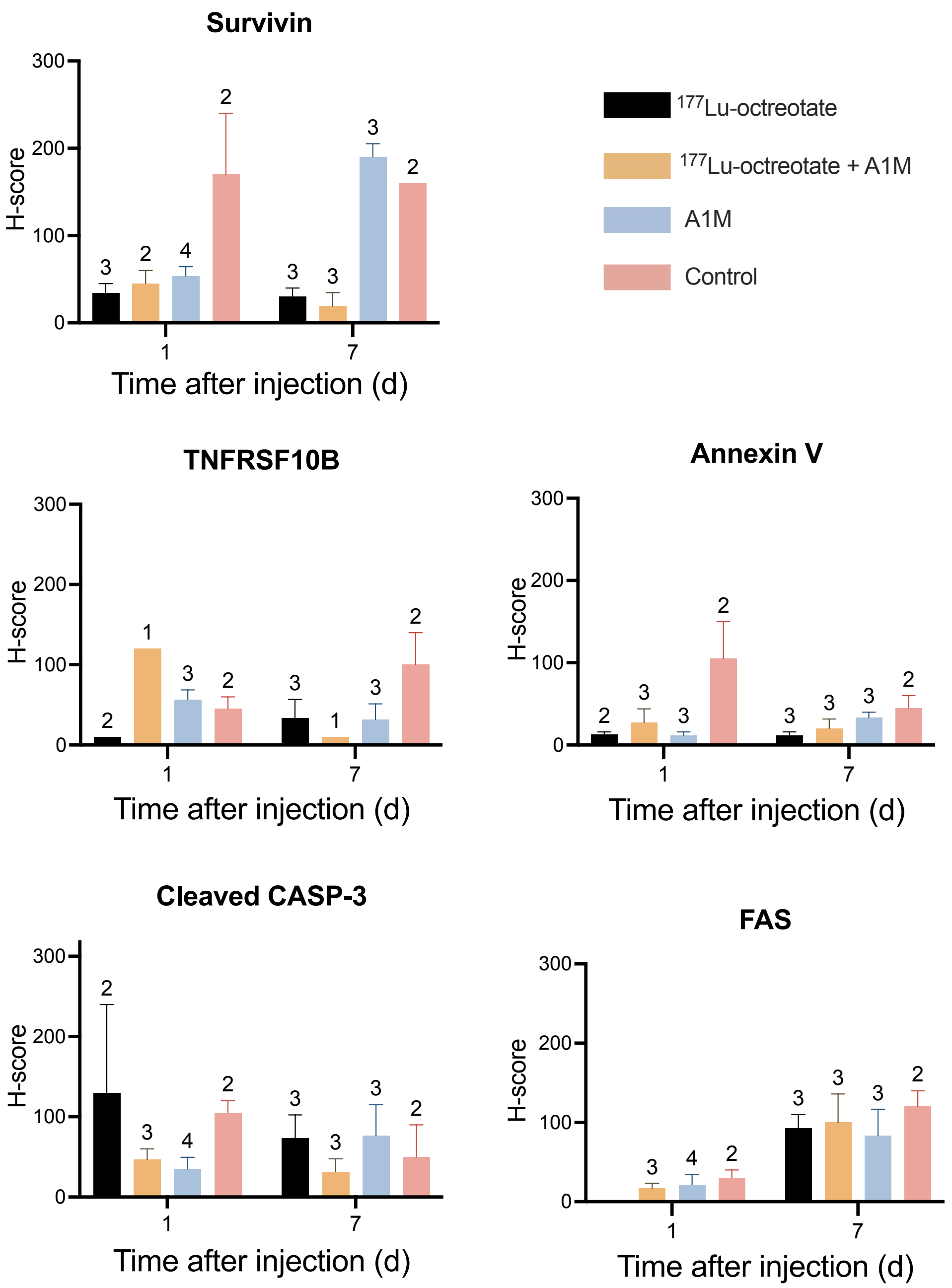


**Supplementary Figure 6.** Histological score (H-score) from immunohistochemical analyses of GOT1 tumors from mice injected with $^{177}$Lu-octreotate, $^{177}$Lu-octreotate + A1M, A1M and saline (controls), and sacrificed after one and seven days. The tumor sections were stained against FAS (**A**), annexin V (**B**) cleaved CASP-3 (**C**), TNFRSF10B (**D**), and survivin (**E**). Error bars represent SEM and sample size is stated above each bar. There were no statistically significant differences between any of the groups. Statistical analysis and illustrations were performed using GraphPad Prism version 9.3.1 for macOS (https://www.graphpad.com/scientific-software/prism/).
